# Production and Formulation of *Alcanivorax borkumensis SK2* Cell Powders for Marine Oil Spill Bioremediation

**DOI:** 10.1002/bab.70009

**Published:** 2025-07-07

**Authors:** Élisabeth Perreault, Denis Groleau, Patrick Vermette

**Affiliations:** ^1^ Laboratoire de bio‐ingénierie et de biophysique de l'Université de Sherbrooke, Department of Chemical and Biotechnological Engineering Université de Sherbrooke Sherbrooke Quebec Canada; ^2^ Department of Chemical and Biotechnological Engineering Université de Sherbrooke Sherbrooke Quebec Canada

**Keywords:** *Alcanivorax borkumensis SK2*, cryoprotectant, differential scanning calorimetry (DSC), freeze‐drying, growth substrate, spray‐drying

## Abstract

Oil spills pose severe threats to marine ecosystems and coastal communities. *Alcanivorax borkumensis SK2*, a marine bacterium with superior hydrocarbon‐degrading capabilities, emerges as a promising agent for bioremediation. This study identified an economical growth substrate for *A. borkumensis SK2* and led to highly viable cell powder formulations for effective applications in contaminated marine environments. Various non‐hydrocarbon substrates were evaluated to replace the costly sodium pyruvate, revealing that canola oil and sunflower oil gave biomass levels (optical density) four times higher than sodium pyruvate (20 ± 2 and 20 ± 1, compared to 4.6 ± 0.4, respectively). Freeze‐drying and spray‐drying approaches were investigated to produce a viable cell formulation. Two screening campaigns of potential freeze‐drying cryoprotectants showed that the proprietary blend of Proventus Bioscience Inc. (Proventus) and 0.5 M glutamate ensured the highest viability, with 2 ± 1×10¹⁰ and 1.1 ± 0.3 × 10¹⁰ CFU/g, after the first screening, and 1.0 ± 0.5 × 10¹⁰ and 6 ± 2 × 10⁹ CFU/g after the second screening. Differential scanning calorimetry (DSC) analysis demonstrated a 9%–15% reduction in ice formation with cooling rates from 5 to 10°C/min. Glutamate reduced ice formation by 5%–9% compared to Proventus’ solution. To promote cell viability during *A. borkumensis SK2* freezing and freeze‐drying, the best product temperatures were determined to be −65°C with 0.5 M glutamate and −59°C with Proventus’ blend. Spray‐drying resulted in cell powders with a viability up to 1.0 ± 0.7 × 10⁵ CFU/g, considerably lower than the levels obtained by freeze‐drying, indicating some potential but also the need for further research and optimization.

## 1 Introduction

From 1970 to 2022, approximately 5.88 million tons of oil were spilled from ships transporting oil [1]. Such spills have devastating consequences on marine ecosystems and coastal populations. The 2002 Prestige oil spill released 63,000 tons of oil near Galicia in Spain [1]. This incident led to the complete loss of all mollusk species on affected beaches and a significant decline in polychaete species [2]. In 2010, the explosion on the Deepwater Horizon drilling rig spilled 4.9 million barrels of crude oil into the Gulf of Mexico [3]. This incident had moderate to severe consequences on the coral nearby [4].

To address such environmental disasters, oil spill remediation techniques have been developed, broadly categorized into three groups. The physical methods, such as booms [5] and skimmers [6], focus on controlling the spread of oil without altering its composition. Often used with physical approaches, chemical methods involve dispersants helping breaking down oil into smaller droplets, facilitating natural microbial degradation [7]. The last is bioremediation, where microorganisms metabolize oil into biomass, carbon dioxide, water, and heat [8], and is garnering increasing interest due to its ecological potential.

One prominent microbial candidate is *Alcanivorax borkumensis SK2*, a marine bacterium known for its strong ability to degrade alkanes [9]. Following an oil spill, its population greatly increases, even becoming dominant [10–12]. *A. borkumensis SK2* metabolism is centered on the conversion of hydrocarbons into energy [13, 14]. Indeed, in the presence of alkanes as the sole source of carbon and energy, *A. borkumensis SK2* can upregulate the enzymes responsible for their metabolization [13].

*A. borkumensis SK2* can degrade a variety of hydrocarbons, including linear alkanes with carbon chain length from C5 to C33 [15], branched alkanes [16], BTEX, motor oils [17], diesel, biodiesel, rapeseed oil [18], aliphatic hydrocarbons [19], as well as alkylated and non‐alkylated polycyclic aromatic hydrocarbons, excluding naphthalene compounds [20].

The general goal of this study was to develop a cost‐effective process to produce highly viable *A. borkumensis SK2* powders. This bacterium was selected to meet the technological needs of our industrial partner, who aims to transform it into a commercially successful product for bioremediation and for its broad versatility in hydrocarbon degradation. Unlike most existing research on *A. borkumensis SK2*, which focuses on the production and formulation of its enzymes involved in hydrocarbon degradation, this study presents a novel approach aimed at directly producing the bacterium itself [17, 21, 22, 23].

The primary specific objective was to identify a cost‐effective growth substrate for *A. borkumensis SK2*. The current method, utilizing sodium pyruvate, though effective, is costly. By identifying a more economical alternative substrate that maintains or enhances biomass yield, the production process can become more viable. Sodium pyruvate is one of the rare non‐hydrocarbon substrates for *A. borkumensis SK2* growth [19, 24]. Among the potential alternatives are formate [19], acetate [19, 24], propionate [19, 24], methylpyruvate and α‐ketoglutarate [19], α‐, β‐ and γ‐hydroxybutyrates [19], as well as peptone [25].

The second specific objective was to develop unit operations for producing *A. borkumensis SK2* powders with high viability, exploring processes such as freeze‐drying and spray‐drying. Formulating *A. borkumensis SK2* into a highly viable powder is necessary for its commercialization and applications. To date, no study has focused on the powdered production of *A. borkumensis SK2*. By addressing the challenges of substrate cost, use of cryoprotectants during freeze‐drying, and dry cell powder production by spray‐drying, we aimed to facilitate the commercialization of *A. borkumensis SK2* for various environmental applications.

## 2 Materials and Methods

### 2.1 Bacterial Strain and Culture Maintenance

*A. borkumensis SK2* was acquired from the German Culture Collection (DSMZ No. 11573, Braunschweig, Germany). Before inoculum preparation, the bacterial culture was carefully maintained at a constant temperature of 4 ± 1°C (for a maximum of 2 weeks) to ensure viability and stability between experiments.

### 2.2 Growth Medium Composition and Sterilization

The liquid growth medium used for all experiments was composed of 23 g NaCl (Fisher Scientific), 1.47 g CaCl_2_ (Fisher Scientific), 0.89 g Na_2_HPO_4_·7H_2_O (Fisher Scientific), 5 g KNO_3_ (Sigma‐Aldrich), 0.10 g FeSO_4_·7H_2_O (Fisher Scientific) and 12 g MgSO_4_·7H_2_O (Fisher Scientific) per liter of distilled water. This mix was inspired from the 809 Medium (DSMZ, Braunschweig, Germany). Care was taken to add each component individually and allow sufficient time for complete dissolution, thus minimizing the risk of precipitation. The medium was subjected to sterilization at 121°C for 40 min.

Additionally, agar plates were employed to assess viability. Agar plates were composed of 23 g NaCl, 1.47 g CaCl_2_, 0.89 g Na_2_HPO_4_·7H_2_O, 5 g KNO_3_, 0.10 g FeSO_4_·7H_2_O, 12 g MgSO_4_·7H_2_O, and 10 g of agar (Fisher Scientific) per liter of distilled water. To ensure appropriate pH, the medium was carefully adjusted to 7.5 using an NaOH (Fisher Scientific) solution. The medium was subjected to the same rigorous sterilization process, 121°C for 40 min.

### 2.3 Screening of Growth Substrates for *A. borkumensis SK2*

#### 2.3.1 Substrate Selection and Preparation

To investigate the metabolic versatility of *A. borkumensis SK2*, seven substrates were selected. Sodium acetate trihydrate (Fisher Scientific) was used as a 40% w/v stock solution, sodium pyruvate (Fisher Scientific) as a 10% w/v stock solution, and peptone (Fisher Scientific) as a 10% w/v stock solution. All these solutions were filtered through a 0.2‐micron filter before use. The study also included the evaluation of heavy mineral oil USP (United States Pharmacopeia norm) (Personnelle trademark, Jean‐Coutu), light mineral oil USP (Fisher Scientific), sunflower oil (SOLEIL D'OR), and canola oil (Compliments trademark, IGA [Sobeys]), which were all autoclaved for 40 min at 121°C.

#### 2.3.2 Experimental Setup

Each condition entailed 100 mL cultures in 500 mL Erlenmeyer flasks with baffles. Following preparation and sterilization of the culture medium, the respective substrates were added to the flasks. Each flask was inoculated with a 3% v/v inoculum of an *A. borkumensis SK2* culture grown on sodium pyruvate (1% w/v) for 3 days at 30°C and 240 rpm.

Subsequently, the 15 flasks for each of the two screening campaigns were incubated at 30°C and 240 rpm for 7 days. During incubation, three samples were collected from each flask at regular intervals, and their absorbance was measured three times at 600 nm, with distilled water as the blank. Manual homogenization was done between each measurement to obtain the most representative result possible, even in the presence of aggregates and foam, which are commonly observed in these cultures.

### 2.4 Cryoprotectant Screening #1

#### 2.4.1 Cryoprotectants Selection and Preparation

A representative cryoprotectant from each category, namely glutamate (amino acid), mannitol (sugar alcohol), and betaine (osmolyte), was chosen. These cryoprotectants were tested at two different concentrations, 0.5 and 0.05 M, to evaluate the impact of concentration on viability after freeze‐drying. For sample preparation, D‐mannitol (Fisher Scientific) (later referred to as mannitol) solutions were prepared at concentrations of 0.1 and 1 M, as well as L‐glutamic acid monosodium salt monohydrate solutions (Fisher Scientific) (later referred to as glutamate) at 0.1 and 1 M. To prevent a potential osmotic shock, these solutions also contained 1% w/v NaCl (Fisher Scientific) and underwent sterilization by autoclaving at 121°C for 30 min.

Likewise, betaine monohydrate (Acros Organic) (later referred to as betaine) solutions at 0.1 and 1 M were prepared, each also containing 1% w/v NaCl. To ensure sterility, these solutions were filtered through 0.2 µm filters. Additionally, the double‐concentrated cryoprotective solution from Proventus was prepared and sterilized according to Proventus’ practices, for comparison purposes. The composition of the Proventus’ cryoprotective solution is, however, a trade secret.

#### 2.4.2 Bioreactor Production and Formulation

A 50 L bioreactor (Micro‐Giant Bioengineering Co. LTD, model MGB‐SV50) with a working volume of 10 L was used and inoculated at 3% (v/v). The inoculum was a 1% (w/v) canola oil *A. borkumensis SK2* culture incubated for 5 days at 240 rpm and 30°C.

The bioreactor was equipped with a programmable logic controller (PLC) system and sensors to manage temperature, dissolved oxygen, pH, airflow, foam formation, agitation speed, and pressure. The PLC control was integrated with a supervisory control and data acquisition (SCADA) system for automatic parameter control. The agitation shaft was mounted with tree Rushton turbines (*∅* = 126 mm). Calibration of the oxygen sensor was conducted in two steps: First, with N_2_ to achieve 0% saturation, and second, with air to achieve 100% saturation. The pH probe (Hamilton) underwent a thorough calibration process using pH 4 and pH 7 buffers (Fisher Scientific). All probe and sensor calibrations were completed prior to sterilization.

The composition of the growth medium was the same as previously described, and the substrate was 1% (w/v) canola oil. To ensure a dissolved oxygen level above 40%, a cascade control system was used to adjust the airflow rate. pH was maintained at 7.5 using a computer‐controlled fermentation setup. The mixing power was 5.6 W/L (400–450 rpm). Water circulation through the bioreactor jacket allowed maintaining the temperature at 30 ± 2°C. The starting air flow rate was 5 L/min, and the pressure was consistently held at 0.1 bar. The silicone‐based antifoam SE‐15 (Sigma‐Aldrich), concentrated at 50 g/L (sterilized at 121°C, 20 min), was used as defoamer, and its addition was done using an automatic foam control system.

For pH regulation, water was sterilized (121°C, 20 min) before adding NaOH (VWR) to achieve a 4 M solution, and the same procedure was followed for the H_3_PO_4_ 2 M (Alphachem Limited) solution. To compensate for water loss during sterilization at 121°C for 30 min, a total of 11 L of growth medium was initially prepared. During fermentation, Gram staining and optical density measurements at 600 nm were conducted to monitor culture purity and evaluate biomass level. A culture sample was taken at the end of the fermentation to assess pre‐lyophilization viability. The culture was harvested and subjected to centrifugation (20,000 × *g*, 20 min, 4°C), and the volume was carefully adjusted with 1% w/v saline to obtain a 20× concentrate. The resulting concentrate was subsequently mixed in equal parts with the previously described cryoprotectant solutions to achieve a final concentration of 10× bacteria and either 0.05 M (C1) or 0.5 M (C2) of the tested cryoprotectants. Additionally, the concentrate was combined in equal parts with Proventus’ cryoprotectant blend. The samples were gently mixed and allowed to sit for 30 min to facilitate interactions between the cryoprotectant and the bacteria. Everything was done in duplicate.

The resulting samples were frozen and lyophilized (Epsilon 2–10D LSC plus, Martin Christ Freeze Dryers) according to the predefined program of Proventus for *A. borkumensis SK2* (Figure S1). The resulting powders were collected, their moisture content measured (Halogen Moisture Analyzer HE73, METTLER TOLEDO) and stored at 4°C for 20 days in closed containers. Afterwards, they were rehydrated with 9 mL of a 1% (w/v) NaCl solution per gram and activated by agitation at 340 rpm for 2 h. A tenfold serial dilution of the resuspended bacteria was prepared using a 1% (w/v) NaCl solution and plated in duplicate. Plates were incubated at 30°C for 5 days, after which colony‐forming units (CFU) were enumerated.

### 2.5 Cryoprotectant Screening #2

Here, the following cryoprotectants were tested: Proventus’ cryoprotective solution and L‐glutamic acid monosodium salt monohydrate (glutamate) at concentrations of 0.05 M (C1), 0.275 M (C2) (newly tested intermediate concentration), and 0.5 M (C3). The other modifications will be specified in the Results and Discussion section.

### 2.6 Differential Scanning Calorimetry Analysis

A 100 mL volume of culture medium containing 1% canola oil was inoculated at 5% (v/v) and incubated at 30°C and 240 rpm for 4.75 days. The culture was subsequently concentrated by centrifugation (20 min at 13,250 × *g*, 4°C), and the pellet resuspended in saline 1% w/v, yielding a 16‐fold increase in biomass concentration. The resulting 16× concentrate was then formulated by mixing it in equal parts with two cryoprotectant solutions:

- L‐Glutamic acid monosodium salt monohydrate (98%, Thermo Scientific) (glutamate) 1 M and 1% (w/v) sodium chloride (Anachemia);
- Proventus’ double‐concentrated cryoprotection blend.

Both cryoprotectant solutions were sterilized at 121°C for 30 min prior to use. The 16× concentrate and the cryoprotectant solutions were also combined in equal parts with a NaCl 1% (w/v) solution, which was also sterilized at 121°C for 30 min. The thermal behavior of each designed mixture was examined using differential scanning calorimetry (DSC) with the DSC 3+ from Mettler Toledo (Schwerzenbach, Switzerland), cooled with liquid nitrogen, based on the heat flux principle. Multi‐point calibration of heat flow and temperature was performed with zinc (Zn), indium (In), water (H_2_O), and *n*‐octane (*n*Oct) as standards; melting points (m.p.) in °C: 419.59 (Zn), 156.60 (In), 0.00 (H_2_O), −56.87 (*n*Oct); specific melting enthalpies in J/g: 104.75 (Zn), 28.31 (In), 336.93 (H_2_O), 175.58 (*n*Oct), Mettler‐Toledo Calibration Kit, ME‐51140313. A calibration check was performed with indium (In). The precise weight of the indium pellet was recorded, and the sample was hermetically sealed in 40 µL aluminum pans (DSC Consumables). An empty, sealed aluminum pan was used as a reference. Between 30 and 40 mg of solutions per pan were used.

DSC analyses were performed in triplicate with three different freezing rates (5°C/min, 10°C/min, 15°C/min) and the same heating rate (10°C/min). The cycles started at 20°C, proceeded to cool to −80°C, held for 5 min, and then proceeded to heat to 20°C. Each sample was subjected to a single cycle.

The same DSC cycles were also applied in triplicate to distilled water to compare its thermal behavior with the tested formulations. However, the final heating temperature of the cycles was increased to fully observe ice melting.

The Midpoint ISO method was employed to determine the temperatures of the glass transitions. In most cases, the glass transition was difficult to observe, and the first derivative of the heat flow curve was used to clearly assess the transition temperature. The onset and endset temperatures were used to describe crystallization. The melting onset temperature was utilized to describe it, while the specific melting enthalpy was employed to evaluate the percentage of crystallinity, with 100% crystallization in distilled water as reference.

### 2.7 Spray‐Drying

Preliminary tests were conducted to select a formulation and parameter sets that would allow the spray‐drying of *A. borkumensis SK2* without excessive nozzle clogging. Approximately 200 mL of a 1% (w/v) canola oil culture medium were inoculated at 5% (v/v) and incubated at 30°C and 240 rpm for 4 days. The culture was then combined with a 30% (w/v) maltodextrin (Sigma Aldrich, dextrose equivalent 4.0–7.0) solution and a 1.93% (w/v) NaCl solution (sterilized at 121°C for 40 min), resulting in mixtures comprising 10% (v/v) *A. borkumensis SK2* culture, 15% (w/v) maltodextrin, and approximately 1% (w/v) NaCl. These mixtures, of 200 mL each, were spray‐dried, while being agitated with a sterilized magnetic stirrer, using a BUCHI Mini Spray Dryer, model B‐290 (Flawil, Switzerland) in a co‐current configuration, with a two‐fluid nozzle. Three sets of operational parameters were tested. Each set of conditions was carried out in duplicate, and before each new set, the spray‐dryer and the nozzle were thoroughly cleaned and sterilized by circulating air at an input temperature of 195°C for 30 min. Between each duplicate, approximately 150 mL of sterile distilled water (sterilized at 121°C for 40 min) were spray‐dried to prevent nozzle clogging and to stabilize parameters before proceeding to the next culture spray‐drying. The resulting powders were collected, their moisture content measured (i‐Thermo G163L), and stored overnight at 4°C. Subsequently, they were rehydrated with 9 mL of a 1% (w/v) saline solution per gram and activated by agitation at 340 rpm for 90 min at 30°C. A 10‐fold serial dilution of the resuspended bacteria was prepared using a 1% (w/v) NaCl solution and plated in triplicate. Plates were incubated at 30°C for 7 days, after which CFU were enumerated.

## 3 Results and Discussion

### 3.1 Screening of Growth Substrates

**FIGURE 1:**
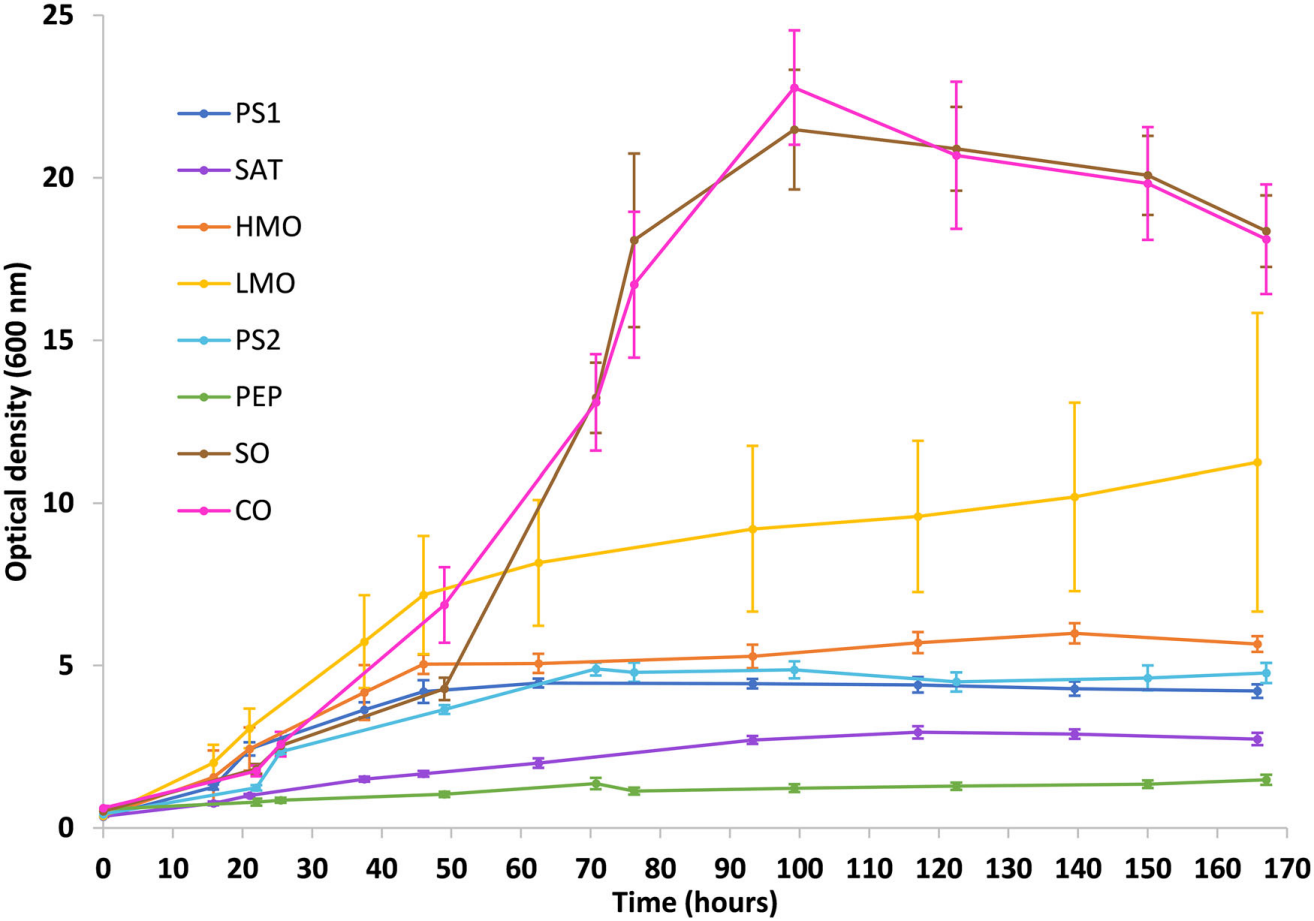
Growth curves of *Alcanivorax borkumensis SK2* on various sources of carbon and energy. The results are means based on data from triplicates, and standard deviations are indicated by vertical bars. PEP: peptone 1% w/v; SAT: sodium acetate trihydrate 1% w/v; PS1: sodium pyruvate 1% w/v first screening; PS2: sodium pyruvate 1% w/v second screening; HMO: heavy mineral oil USP 1% w/v; LMO: light mineral oil USP 1% w/v; SO: sunflower oil 1% w/v; CO: canola oil 1% w/v.

Canola oil and sunflower oil emerged as the two most promising substrates for subsequent work. The optical densities obtained with these substrates were approximately four times higher than those achieved with sodium pyruvate (Figure 1). Specifically, after 150 h, sodium pyruvate yielded an optical density of 4.6 ± 0.4, while canola oil and sunflower oil resulted in optical densities of 20 ± 2 and 20 ± 1, respectively.

To date, no substrate capable of achieving such high optical densities with *A. borkumensis SK2* has been identified. In the literature, optical densities of up to 1.6 have been reported after 60 h using hexadecane as the sole carbon and energy source [26]. Similarly, optical densities of approximately 1.8 had been observed after 6 days of fermentation with peptone at 500 mg/L [25], similarly to our results after 167 h. Regarding the other substrates, sodium acetate yielded an optical density of 2.7 ± 0.2 after 167 h, while heavy and light mineral oil resulted in optical densities of 5.7 ± 0.2 and 11 ± 5, respectively.

Both sunflower oil and canola oil gave similar growth curves, with comparable standard deviations (Figure 1). Furthermore, sunflower oil and canola oil have very similar molar masses, with values of 876 g/mol [27] for sunflower oil and 877 g/mol [28] for canola oil. Therefore, both sets of replicates received an equivalent amount of oil at the molecular level, making it inappropriate to differentiate between these two substrates based on molecular mass alone.

However, it is worth noting that sunflower oil is at least twice as expensive as canola oil. Indeed, the wholesale price per ton of canola oil is approximately $2585 USD [29], whereas the price for sunflower oil is approximately $6190 USD per ton [30]. Therefore, canola oil was selected for the subsequent studies as it brings a significant reduction in substrate cost. The wholesale price per ton of sodium pyruvate 99% was $13,716 USD [31], so the substrate price reduction is approximately 88%, or a four‐fold decrease in substrate cost. This analysis is only for comparison purposes, and proper cost analysis should be performed with pricing adapted to industrial‐scale production.

### 3.2 Cryoprotectant Screening #1

#### 3.2.1 Fermentation #1

**FIGURE 2:**
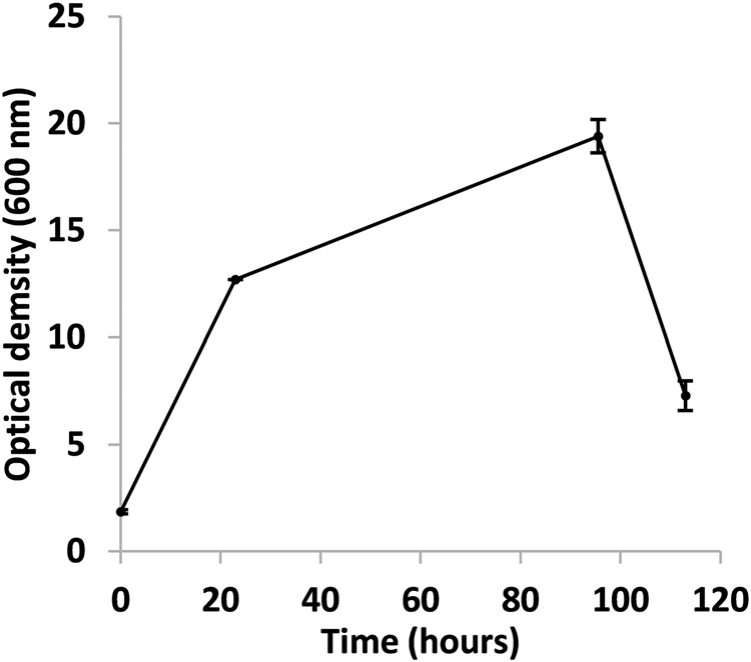
Growth curve of *Alcanivorax borkumensis SK2* on 1% (w/v) canola oil as the sole carbon and energy source in a 50‐L bioreactor with a working volume of 10 L (Fermentation #1). Results are means from triplicates, with standard deviations indicated by vertical bars. The 10 L used did not prohibit effective mixing and measurements of pH, dissolved oxygen, etc.

**FIGURE 3:**
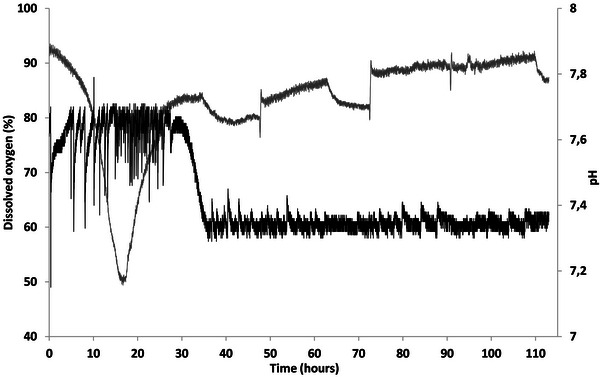
Monitoring of dissolved oxygen and pH during Fermentation #1. Gray curve, dissolved oxygen; black curve, pH.

Growth was faster in the bioreactor (Figure 2). In flasks, the lag phase lasted approximately 24 h, followed by an exponential phase of about 72 h (Figure 1). In contrast, the bioreactor data showed a drop in dissolved oxygen right at the start of the fermentation (Figure 3), suggesting the absence or a very short lag phase. This difference could be attributed to several factors, such as improved mass and heat transfer dynamics favoring oxygen transfer and solubilization. *A. borkumensis SK2* has been described as particularly sensitive to low oxygen levels [22]. A recent study has also observed that this bacterium grew faster in a bioreactor [24]. More effective mixing and improved substrate emulsion in the bioreactor were proposed as reasons for this. In our case, this likely resulted in smaller canola oil droplets, increasing the contact surface between the bacteria and the substrate, as observed in the previously mentioned study.

Moreover, it is noteworthy that the culture underwent an interesting transition after 36–40 h of fermentation (Figure 3), suggesting a shift to the stationary state. Similar findings were observed in a 150‐L bioreactor using 5% (v/v) motor oil as substrate, where the decrease in dissolved oxygen concentration correlated with the exponential phase, and growth stabilization after 40 h was associated with a transition to the stationary phase [22]. The drop in optical density at the end of the fermentation process could be attributed to several factors: wall growth, excessive foaming, and/or cell aggregation [24].

#### 3.2.2 Post‐Lyophilization #1

**FIGURE 4:**
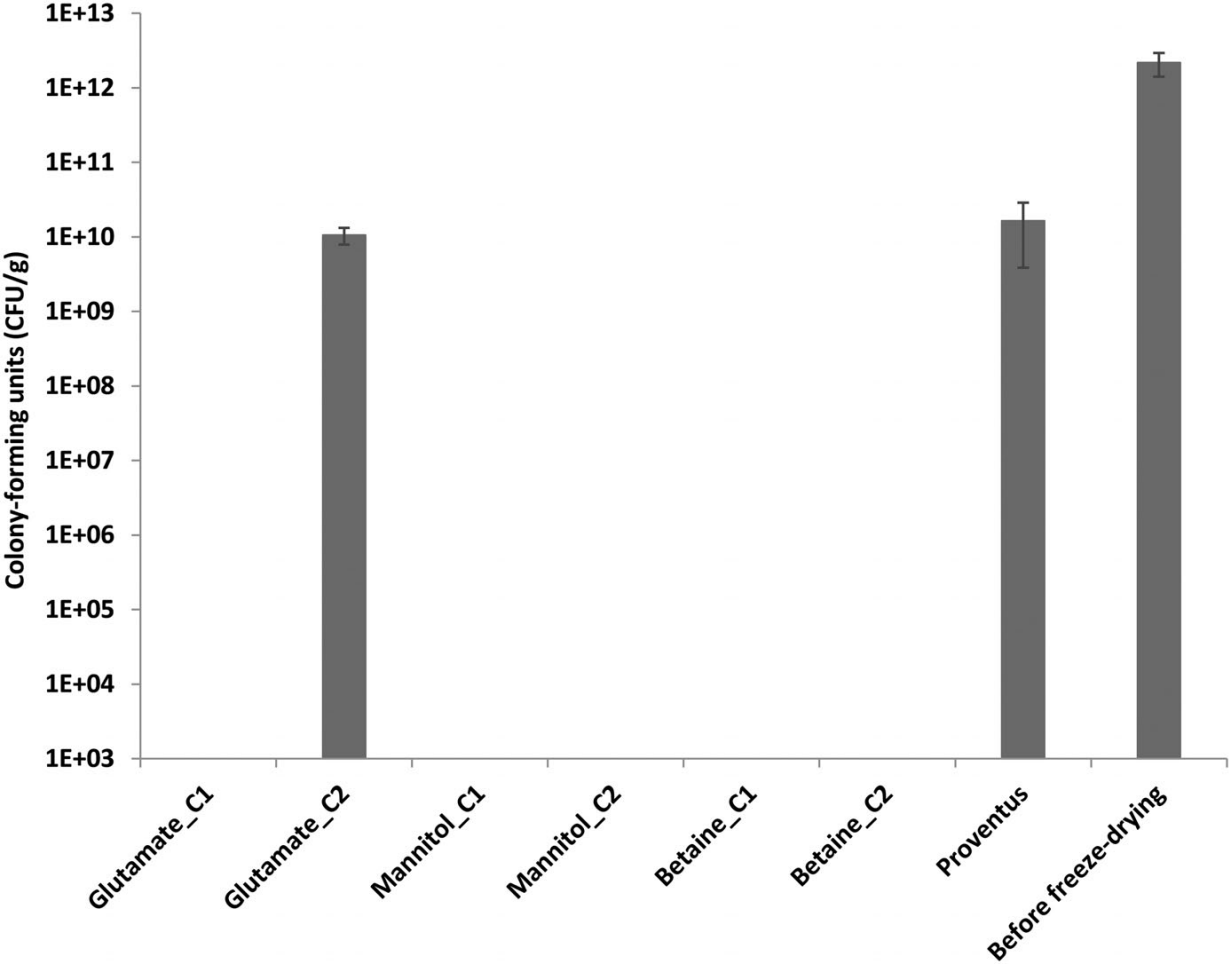
Viability of *Alcanivorax borkumensis SK2* after freeze‐drying with different cryoprotectants at concentrations of 0.05 M (C1) and 0.5 M (C2), compared to Proventus’ cryoprotectant blend. The absence of a vertical bar indicates that the bacterial count was below the detection limit, as plating was initiated from the 10⁻⁵ dilution. The results represent the means obtained from duplicates, with standard deviations indicated by vertical bars.

The cryoprotectants were selected based on scientific literature, approximate cost, and specific properties. To the best of our knowledge, no study has been conducted on the freeze‐drying of *A. borkumensis SK2* with cryoprotectants. Therefore, a representative cryoprotectant from each category was tested. Figure 4 illustrates the protective effect of these four cryoprotectants at concentrations C1 (0.05 M) and C2 (0.5 M). Glutamate C1 (0.05 M), mannitol C1 (0.05 M), mannitol C2 (0.5 M), betaine C1 (0.05 M), and betaine C2 (0.5 M) did not support the survival of *A. borkumensis SK2* or resulted in survival rates too low to be detectable after lyophilization. Conversely, Proventus’ cryoprotective blend and Glutamate C2 demonstrated by far the highest survival rates, with viable counts of 2 ± 1×10¹⁰ and 1.1 ± 0.3×10¹⁰ CFU/g, respectively. These findings strongly suggested that a higher concentration of glutamate is highly favorable for *A. borkumensis SK2* survival, as Glutamate C1 gave no or low viability compared to Glutamate C2. Therefore, further investigations to test the effect of intermediate concentrations of glutamate between C1 and C2 on bacterial survival were performed. These results are presented under Cryoprotectant Screening #2. Comparatively, 15% (w/v) lactose and 10% (w/v) mannitol were tested as single cryoprotectants for another marine bacterium, *Pseudoalteromonas nigrifaciens*, yielding survival rates of 17.05% and 17.53%, respectively [32]. So, mannitol was beneficial for *P. nigrifacens* but not so much for *A. borkumensis SK2*. This highlights the species‐dependent nature of cryoprotectants.

The residual moisture level of *A. borkumensis SK2* powders, after freeze‐drying using various cryoprotectants at two different concentrations, ranged from 1.5% ± 0.5% to 1.84% ± 0.06% for the C1 (0.05 M) concentration (Figure S2). The Mannitol C2 (0.5 M) formulation displayed the lowest residual moisture content, 0.88% ± 0.05%, followed by the betaine C2 formulation, with 1.04% ± 0.01% moisture. Conversely, the Glutamate C2 formulation showed the highest residual moisture, 4.3% ± 0.3%. Prior research suggested that a residual moisture content of 2.8%–5.6% promoted *Lactobacillus salivarius* survival during storage [33], while another study found that 5% residual moisture was optimal for preserving *Lactobacillus acidophilus* for 3 months at 37°C [34]. Thus, residual moisture levels obtained for *A. borkumensis SK2* appear suitable for long‐term storage. Stability tests over weeks or even months will be necessary to determine the exact influence of these residual moisture levels on viability.

### 3.3 Cryoprotectant Screening #2

In the first lyophilization experiment, the cryoprotectants, namely 0.5 M glutamate and Proventus’ proprietary blend, demonstrated promising cell survival. Furthermore, growth was observed to be faster in the bioreactor compared to flasks, suggesting that the harvesting occurred during the stationary phase. The time of harvest may influence cell survival after lyophilization, as different bacteria respond differently [35]. Therefore, this second freeze‐drying trial included an intermediate concentration of glutamate (0.275 M) alongside the previously tested concentrations of 0.5 and 0.05 M, as well as Proventus’ cryoprotectant blend for comparison purposes, together with evaluating the effect of harvesting at the end of the exponential phase on post‐lyophilization cell viability. To evaluate the effect of culture phase on viability, the fermentation period was shortened to 41 h from the original 113 h to enable harvesting at the end of the exponential phase, rather than during the stationary phase.

Additionally, the role of glutamate concentration on cell viability was rigorously investigated using smaller plating dilutions for the C1 (0.05 M) concentration to better understand the impact of glutamate concentration on viability. This was particularly important given the absence of viable cells for the C1 concentration in the first trial, where only dilutions from 10⁻⁵ were plated. The initial goal was to screen for cryoprotectant types and approximate effective concentrations that could support a viable production bioprocess. This follow‐up trial aimed to refine the concentration range to enhance cell survival after freeze‐drying.

#### 3.3.1 Fermentation #2

**FIGURE 5:**
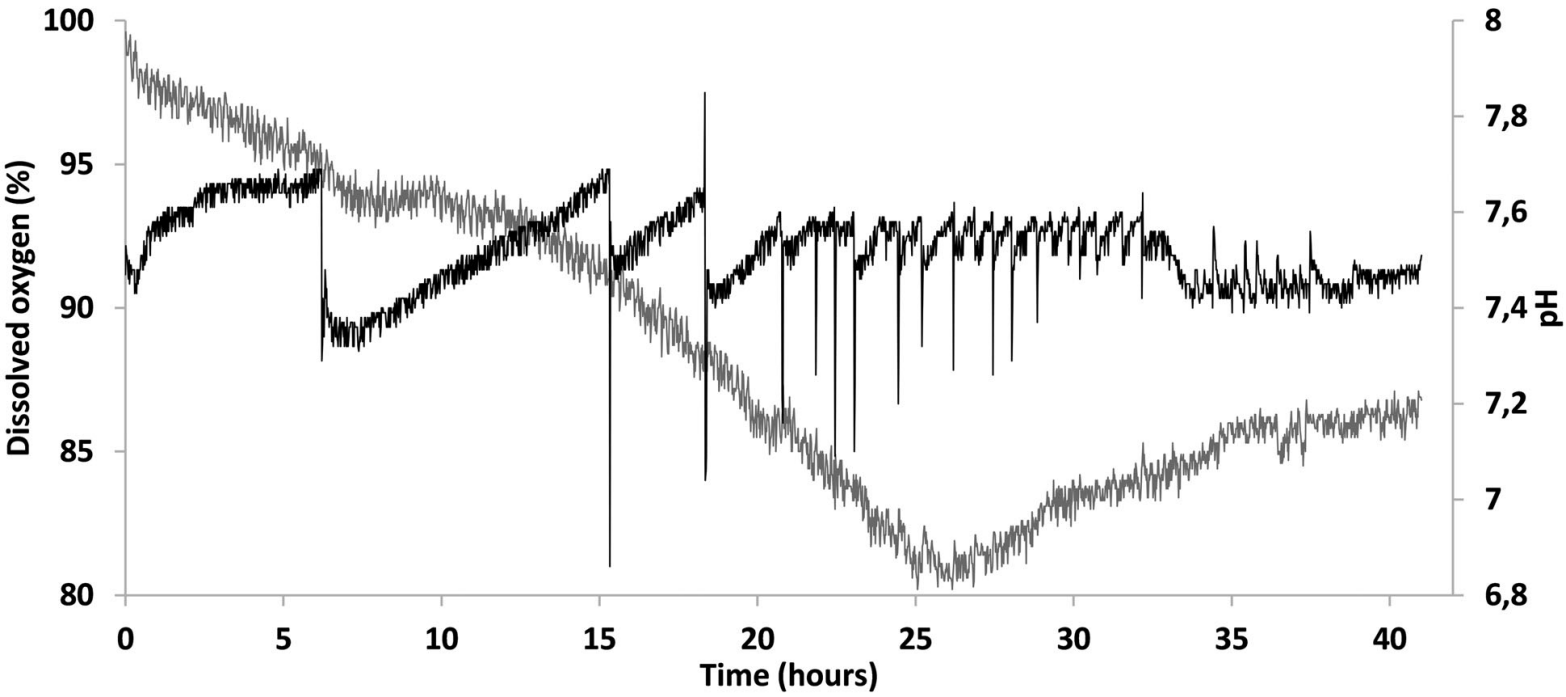
Dissolved oxygen and pH profiles (Fermentation # 2). Gray curve, dissolved oxygen; black curve, pH.

In the first fermentation, the pH began to drop at 32 h and stabilized between 7.3 and 7.4 at 36 h (Figure 3). In comparison, the pH in the second fermentation dropped slightly between 32 and 33 h and then stabilized between 7.4 and 7.5 (Figure 5). So, the pH profile in both fermentations was similar, suggesting that the exponential phase likely ended at around the same time. The optical density measurements must be seen as semi‐quantitative results. The reason for this is the partial hydrophobic nature of *A. borkumensis SK2*, which facilitates its access to the oil–water interface by reducing the interfacial tension when in contact with a hydrophobic substrate [36]. This interaction initiates the synthesis of a biofilm, forming clusters of various sizes held together by extracellular polysaccharides, leading to the formation of aggregates [36]. Biofilm formation and flocculation, possibly caused by biofilm clusters, have been previously observed with a hydrophobic substrate in stirred‐tank bioreactors [24]. This phenomenon appeared to have occurred during the fermentation, albeit more intensely than during the first fermentation. Indeed, the rheological behavior of the culture and the resulting cell concentrate was distinctive. The cells exhibited high hydrophobicity, causing rapid separation from the aqueous phase (Figure S3). The concentrate was even challenging to homogenize due to *A. borkumensis SK2's* tendency to aggregate and form flocs, resulting in a highly elastic behavior (seemed to hold together as one and regained its form instantly after deformation). Therefore, to achieve an even distribution of bacteria in the freeze‐dried samples, a 16× concentrate was used instead of a 20× concentrate.

**FIGURE 6:**
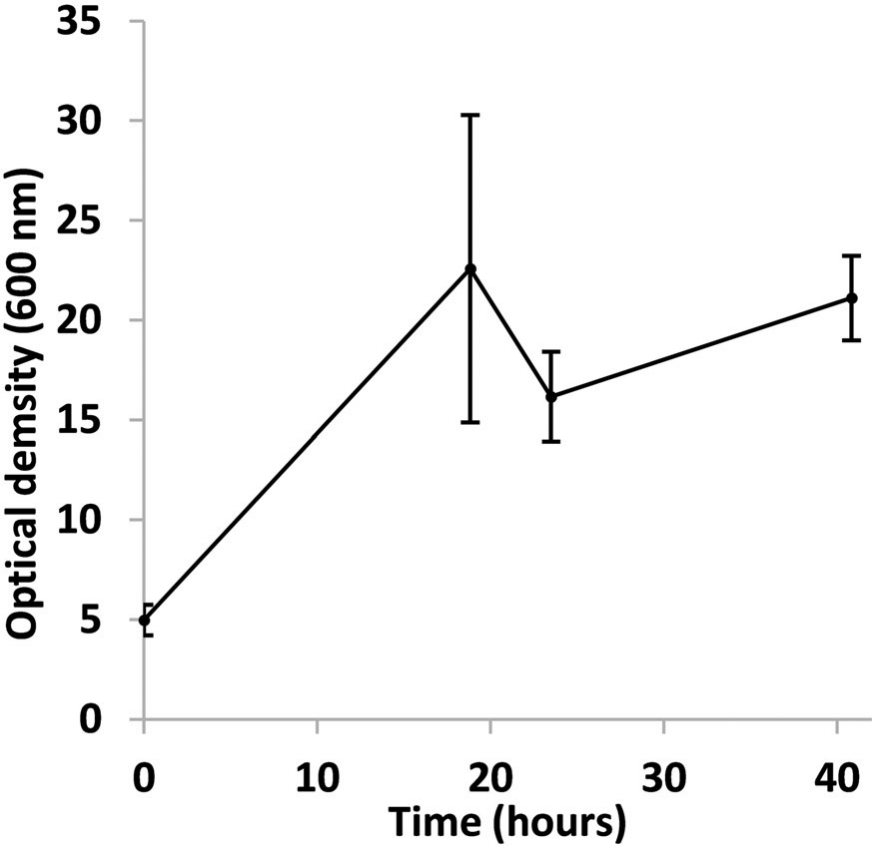
Growth curve of *Alcanivorax borkumensis SK2* on 1% (w/v) canola oil as the sole carbon and energy source in a 50‐L bioreactor with a working volume of 10 L (Fermentation #2). An alternative defoamer was used, 5% polypropylene glycol 2000 (Dow) solution autoclaved at 121°C for 20 min due to a supply shortage. Results are means from triplicates, with standard deviations indicated by vertical bars.

Finally, it can be concluded that a cultivation time longer than 40–41 h does not seem to enable greater production of *A. borkumensis SK2* cells (Figure 6). As a result, fermentation duration is no longer the bottleneck of the newly proposed process.

#### 3.3.2 Post‐Lyophilization #2

**FIGURE 7:**
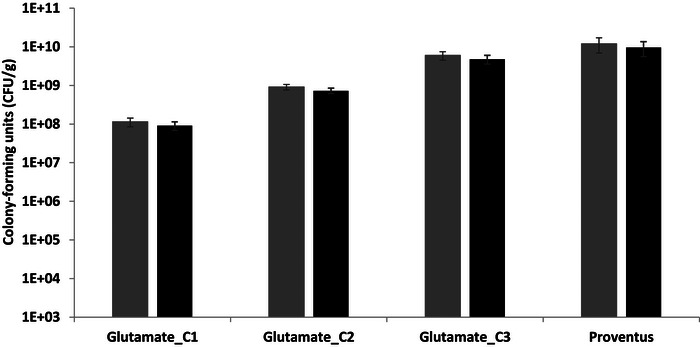
Viability of *Alcanivorax borkumensis SK2* after freeze‐drying with glutamate at concentrations of 0.05 M (C1), 0.275 M (C2), and 0.5 M (C3), compared to Proventus’ cryoprotectant blend. The samples were stored for 21–24 days at 4°C before rehydration. Gray bars, results adjusted for 20× concentrate (previously used culture concentration); black bars, non‐adjusted results (16× concentrate, current concentration). The adjustment for a 20× concentrate was performed by multiplying the viability results by 20 and dividing by 16. Data represent means obtained from duplicate, with standard deviations indicated by vertical bars.

As clearly shown in Figure 7, Glutamate C1 (0.05 M) is not suitable for supporting the production of a highly viable *A. borkumensis SK2* powder following freeze‐drying. However, Glutamate C3 was much more promising, essentially almost as good as Proventus’ cryoprotectant blend with 5 ± 1 × 10⁹ and 1.0 ± 0.4 × 10¹⁰ CFU/g, respectively.

During this trial, the rheological behavior of the culture and its concentrate exhibited unique characteristics, as previously mentioned. The rehydrated bacteria exhibited a similar behavior, forming flocs and sedimenting within seconds, which likely resulted in an underestimation of post‐lyophilization viability. Given these observations, glutamate 0.5 M and Proventus’ blend appeared to be the most promising cryoprotectants for further studies, based on both these new and previous results.

Regarding moisture levels, the formulation containing Glutamate C2 (0.275 M) exhibited the highest residual moisture content (2.40%), followed by C3 (0.5 M) (1.65%), C1 (0.05 M) (1.32%), and Proventus’ blend (0.90%) (Figure S4). As previously mentioned, these moisture levels fall within the acceptable range for storing freeze‐dried *A. borkumensis SK2* powders.

### 3.4 DSC Analysis

The operating temperature influences the viability of microorganisms during lyophilization in several ways [37]. First, if freezing, the first step of lyophilization, is performed at a higher temperature than the bacterial glass transition temperature (*Tg'*), the cells remain active [38], allowing water to diffuse from the intracellular environment to the extracellular environment, increasing ice formation and cell dehydration. Excessive dehydration may cause damage to cellular structures and lead to additional mortality [37].

Second, it is crucial to preserve the bacterial glassy state and its freeze‐concentrated solution (FCS) during primary drying in lyophilization [37, 39]. This is achieved by selecting lyophilization operating parameters (pressure and shelf temperature) that keep the product temperature always below the *Tg'* [40]. Lyophilization at a higher temperature than the *Tg'* can cause osmotic exchanges between the cells and their immediate environment [38]. This leads to significant water loss and avoidable additional mortality, as the cells are exposed to an increasingly concentrated environment during primary drying [38].

Therefore, selecting an appropriate freezing and primary drying temperature is essential to optimize viability in lyophilization. This temperature must be lower than the microorganism *Tg'* to avoid the adverse effects described above. In this regard, DSC tests were undertaken to determine the glass transition temperature of *A. borkumensis SK2* suspensions and to assess the impact of freezing rate on thermal phenomena. Additionally, the influence of cryoprotectants on the thermal behavior of *A. borkumensis SK2* 8× suspensions, and vice versa, was investigated.

#### 3.4.1 Crystallization and Melting Analysis

DSC data analysis facilitated the determination of crystallization and melting events for various formulations of *A. borkumensis SK2* at three different freezing rates, as well as for the cryoprotectants alone. Reducing the freezing rate resulted in an increase in the freezing point of the products, whatever its composition (Table 1). However, freezing is a complex crystallization phenomenon, influenced by both kinetics and thermodynamic factors such as nucleation and cohesive energy [41]. Due to this complexity, the cooling curves obtained for all samples did not reveal a clear trend regarding the precise effect of the presence or absence of cryoprotectants on the crystallization behavior of *A. borkumensis SK*2 suspensions. This lack of trend is attributed to the unpredictable kinetic phenomena involved.

**TABLE 1:** Melting and crystallization temperatures of a *Alcanivorax borkumensis SK2* 8× concentrate in the presence and absence of cryoprotectants, as well as cryoprotectants alone, under various freezing rates.

Formulation	Freezing rate	Melting #1 (onset) (°C)	Specific melting enthalpy #1 (J/g)	Melting #2 (onset) (°C)	Specific melting enthalpy #2 (J/g)	Primary crystallization (onset) (°C)	Primary crystallization (endset) (°C)
8× AB^a^	15°C/min	−3.3 ± 0.3	245 ± 15	−26.5 ± 0.3	2.4 ± 0.5	−21 ± 5	−32 ± 3
	10°C/min	−3.6 ± 0.1	243 ± 11	−26.5± 0.1	2.3 ± 0.2	−20 ± 2	−26 ± 1
	5°C/min	−3.6 ± 0.1	255 ± 6	−26.4 ± 0.3	2.0 ± 0.6	−18 ± 2	−21 ± 1
8× AB + Glu^a^	15°C/min	−7.5 ± 0.2	205 ± 3	N/A	N/A	−24.3 ± 0.4	−35 ± 1
	10°C/min	−7.9 ± 0.2	207 ± 5	N/A	N/A	−21 ± 3	−28 ± 2
	5°C/min	−7.5 ± 0.3	205 ± 3	N/A	N/A	−17 ± 3	−21 ± 2
Prov + 8× AB^a^	15°C/min	−4.1 ± 0.4	224 ± 18	N/A	N/A	−23 ± 1	−33 ± 2
	10°C/min	−4.2 ± 0.2	226 ± 9	N/A	N/A	−18 ± 1	−25 ± 1
	5°C/min	−4.1 ± 0.1	232 ± 1	N/A	N/A	−20 ± 1	−22 ± 1
Glu^a^	15°C/min	−7.28 ± 0.03	234 ± 5	N/A	N/A	−30 ± 3	−37 ± 2
	10°C/min	−7.42 ± 0.03	237 ± 4	N/A	N/A	−20 ± 5	−26 ± 4
	5°C/min	−7.6 ± 0.1	240 ± 8	N/A	N/A	−17 ± 1	−20 ± 1
Prov^a^	15°C/min	−3.9 ± 0.2	262 ± 10	N/A	N/A	−20 ± 2	−29 ± 2
	10°C/min	−4.0 ± 0.2	265 ± 13	N/A	N/A	−17 ± 3	−22 ± 2
	5°C/min	−4.00 ± 0.03	268 ± 5	N/A	N/A	−17 ± 2	−19 ± 1
Distilled water	15°C/min	3 ± 4	336 ± 11	N/A	N/A	−22.7 ± 0.3	−31 ± 1
	10°C/min	1 ± 1	338 ± 7	N/A	N/A	−22 ± 1	−26 ± 2
	5°C/min	2 ± 1	295 ± 21	N/A	N/A	−19 ± 1	−21.8 ± 0.3

^a^ 8× AB: Eight‐fold concentrated *A. borkumensis SK2* culture with 1% (w/v) sodium chloride; 8× AB + Glu: 0.5 M glutamate with 1% (w/v) sodium chloride and eight‐fold concentrated *A. borkumensis SK2* culture; Prov + 8× AB: Proventus’ proprietary cryoprotective blend with 0.62% (w/v) sodium chloride and eight‐fold concentrated *A. borkumensis SK2* culture; Glu: 0.5 M glutamate with 1% (w/v) sodium chloride; Prov: Proventus’ proprietary cryoprotective blend with 0.62% (w/v) sodium chloride.

“N/A” indicates that no detectable results were obtained for this condition.

The 8× concentrate was the only experimental condition to display two distinct melting events (Figure S5 and Table 1). The second melting event is associated with a secondary crystalline phase. This phase is hypothesized to arise from interactions between the bacteria and water, resulting in a concentrated interphase. The energy of interactions within this interphase is lower than that within water crystals alone, leading to a notably low melting temperature (approximately −26.5°C). Cryo‐SEM analyses could offer further insights into the composition of this secondary crystalline phase. The absence of secondary crystallization, representing this second phase, may be attributed to kinetic phenomena, such as the lack of nucleation sites to generate different crystals. Alternatively, it is plausible that crystallization of this phase occurred during the 5‐min isothermal hold at −80°C.

The disappearance of the second melting event upon the addition of cryoprotectants suggested their role in reducing ice formation within the concentrated liquid phase containing bacteria and other solutes. This reduction likely mitigated mechanical damage.

Adding cryoprotectants to the eight‐fold concentrated *A. borkumensis SK2* suspension (8× AB), regardless of their nature, lowered the melting temperature of the mixtures. This is due to the addition of solutes to the aqueous phase, which causes a depression of the melting temperature [42]. The same phenomenon explains the reduced melting temperature of water when bacterial concentrates or cryoprotectants are added. L‐Glutamate is the cryoprotectant that most influenced the melting temperature of the concentrated bacterial suspensions, compared to Proventus's blend. Indeed, it induced a reduction in temperature ranging from 3.9°C to 4.3°C, depending on the cooling rate (Table 1). In contrast, Proventus's blend only lowered the temperature by 0.5°C–0.8°C.

Adding bacteria to the cryoprotectants did not result in significant variations in the melting events, suggesting stronger interactions between the cryoprotectants and water than between the bacteria and water. This is plausible as both cryoprotectants are dissolved in water, while bacteria are dispersed.

*Note*: When evaluating the effect of adding cryoprotectants to the eight‐fold concentrated *A. borkumensis SK2* suspensions (8× AB), the thermal properties of the 8× AB suspensions were compared to those of the 8× AB suspensions combined with a cryoprotectant (Prov + 8× AB or Glu + 8× AB). Conversely, when evaluating the impact of adding 8× AB to a cryoprotectant solution (Prov or Glu), the thermal properties of the cryoprotectant alone were compared to those of its combination with 8× AB (Prov + 8× AB or Glu + 8× AB). This note applies to all DSC analysis results.

#### 3.4.2 Crystallinity Analysis

##### 3.4.2.1 Impact of Cooling Rate on Crystallinity

Regardless of their composition, all tested samples exhibited a reduction in crystallinity when the cooling rate increased from 5°C/min to 10°C/min (Table S1). This increase in cooling rate resulted in a decrease in ice formation ranging from 9% to 15%, depending on sample composition (PROV: 14%, PROV+8× AB: 13%, Glu: 12%, GLU+8× AB: 9%, 8× AB: 15%). Increasing the cooling rate further to 15°C/min only led to a marginal reduction in ice formation, ranging from 0% to 1%. Therefore, a cooling rate of 10°C/min is recommended to minimize ice formation, thus reducing the risk of mechanical damage to bacteria during freezing [40]. Further studies could be conducted to confirm this cooling rate based on cell survival and crystal size [43]. To precisely regulate the cooling rate, freeze‐dryers equipped with internal freezing control capabilities must be used. Through programming specific setpoint temperatures and desired cooling rates, coupled with the use of a product temperature probe, meticulous control over the cooling process could be attained.

##### 3.4.2.2 Impact of 8× AB Concentrate Addition on Cryoprotectants Crystallinity

The addition of the 8× AB concentrate to cryoprotectant solutions resulted in a similar reduction in crystallization for Proventus’ blend and Glutamate C3 (0.5 M). In both cases, adding 8× AB reduced the amount of ice formed by 10%–13% (Table S2). This suggests that both cryoprotectants had energetically similar interactions with the bacteria.

##### 3.4.2.3 Impact of Cryoprotectant Addition on 8× AB Concentrate Crystallinity

Glutamate significantly reduced the amount of ice formed in the *A. borkumensis SK2* 8× concentrate compared to Proventus’ blend. Indeed, it induced a reduction in crystallization of 5%–9% depending on the cooling rate (15°C/min: 5%, 10°C/min: 4%, and 5°C/min: 9%) (Table S2). In contrast, Proventus’ blend had no significant impact on ice formation.

The ability of cryoprotectants to reduce the amount of ice formed is crucial because when water crystallizes, it excludes solutes, thereby increasing solute concentration in the extracellular phase [44, 45]. Therefore, the more effectively a cryoprotectant reduces ice formation, the more effective it is in mitigating mechanical damage [40] and in reducing the ionic concentration to which cells are exposed [45]. Based on these criteria alone, glutamate was the most effective cryoprotectant for *A. borkumensis SK2*.

The greater reduction in ice formation by glutamate compared to the Proventus’ blend could be explained by the presence of amine (NH_2_) and alcohol (OH) functional groups, which form hydrogen bonds with liquid water, thus impeding nucleation and ice development [46]. Indeed, the effectiveness of a cryoprotectant often depends on the number of hydrophilic functional groups it possesses and on the strength of their interaction with water [46].

#### 3.4.3 Glass Transition Analysis

When cooling aqueous solutions below 0°C, three main thermal events occur [39]:

- Water crystallizes into planar dendrites, excluding solutes and concentrating them, along with cells in the remaining aqueous phase called freeze‐concentrated solution (FCS), which is distributed between ice dendrites (FCS1) and outside the dendrites (FCS2);
- FCS1 transitions to a glass state at its specific *Tg*;
- FCS2 transitions to a glass state at its specific *Tg*.

This explanation aligns with the literature [45]. Bacteria have been observed both outside and in between ice crystals [45]. These authors also suggested that bacteria are likely to experience different solute concentrations throughout the FCS, which supports the previous explanation that each FCS has its glass transition temperature depending on its composition [45].

Glass transition is a process occurring in amorphous or semi‐crystalline materials where it transitions between a rigid, glassy state and a more rubbery, flexible state. It may happen in localized areas of a material or within the entire material, depending on its composition. DSC scans and application of the first derivative revealed that 8× AB had a single glass transition, with temperatures ranging from approximately −57.4°C to −60°C under different freezing rates (Table 2). In contrast, 8× AB + glutamate showed first transition at −65°C, independent of the freezing rate, and a second transition between −51°C and −52°C (Figure S6 and Table 2). Proventus's blend + 8× AB exhibited glass transition temperatures similar to 8× AB, with the first transition occurring between −58°C and −59°C and the second between −45°C and −46.1°C. Glutamate alone displayed glass transition temperatures ranging from approximately −62.7°C to −63°C for the first transition and −51°C to −52.1°C for the second transition. Proventus's blend alone showed glass transition temperatures varying from around −53.3°C to −54°C for the first transition and from −38.66°C to −39.0°C for the second transition. Distilled water did not exhibit any glass transition.

**TABLE 2:** Glass transition temperatures and shifts of a *Alcanivorax borkumensis SK2* 8× concentrate in the presence and absence of cryoprotectants, as well as cryoprotectants alone, under various freezing rates.

Formulation	Freezing rate	Glass transition #1 (midpoint ISO) (°C)	Glass transition #2 (midpoint ISO) (°C)	Glass transition #1 shift (midpoint ISO) (°C)	Glass transition #2 shift (midpoint ISO) (°C)
8× AB^a^	15°C/min	−59 ± 1	N/A	N/Ab	N/A
	10°C/min	−57.4 ± 0.5	N/A	N/A	N/A
	5°C/min	−60 ± 1	N/A	N/A	N/A
8× AB + Glu^a^	15°C/min	−65.15 ± 0.03	−51.9 ± 0.2	N/A	N/A
	10°C/min	−65.1 ± 0.3	−52 ± 1	N/A	N/A
	5°C/min	−65 ± 1	−51 ± 1	N/A	N/A
Prov + 8× AB^a^	15°C/min	−59 ± 1	−45 ± 1	N/A	N/A
	10°C/min	−58 ± 1	−45.8 ± 0.1	N/A	N/A
	5°C/min	−58.4 ± 0.1	−46.1 ± 0.1	N/A	N/A
Glu^a^	15°C/min	−62.7 ± 0.4	−52.1 ± 0.2	N/A	N/A
	10°C/min	−63 ± 1	−52 ± 1	N/A	N/A
	5°C/min	−62.9 ± 0.2	−51 ± 1	N/A	N/A
Prov^a^	15°C/min	−53.3 ± 0.5	−38.66 ± 0.02	N/A	N/A
	10°C/min	−53.5 ± 0.2	−39.0 ± 0.3	N/A	N/A
	5°C/min	−54.0 ± 0.4	−39.0 ± 0.2	N/A	N/A
Distilled water	15°C/min	N/A	N/A	N/A	N/A
	10°C/min	N/A	N/A	N/A	N/A
	5°C/min	N/A	N/A	N/A	N/A
8× AB^a^ VS Prov + 8× AB^a^	15°C/min	N/A	N/A	−0.5	N/A
	10°C/min	N/A	N/A	1.1	N/A
	5°C/min	N/A	N/A	−2.0	N/A
8× AB^a^ VS Glu + 8× AB^a^	15°C/min	N/A	N/A	5.9	N/A
	10°C/min	N/A	N/A	7.8	N/A
	5°C/min	N/A	N/A	4.3	N/A
Glu + 8× AB^a^ VS Glu ^a^	15°C/min	N/A	N/A	−2.5	0.2
	10°C/min	N/A	N/A	−2.3	−0.6
	5°C/min	N/A	N/A	−1.8	0.4
Prov + 8× AB^a^ VS Prov^a^	15°C/min	N/A	N/A	−5.4	−6.6
	10°C/min	N/A	N/A	−5.0	−6.8
	5°C/min	N/A	N/A	−4.5	−7.1

^a^ 8X AB: 8‐fold concentrated *A. borkumensis SK2* culture with 1% (w/v) sodium chloride; 8X AB + Glu: 0.5 M glutamate with 1% (w/v) sodium chloride and 8‐fold concentrated *A. borkumensis SK2* culture; Prov + 8X AB: Proventus’ proprietary cryoprotective blend with 0.62% (w/v) sodium chloride and 8‐fold concentrated *A. borkumensis SK2* culture; Glu: 0.5 M glutamate with 1% (w/v) sodium chloride; Prov: Proventus ’proprietary cryoprotective blend with 0.62% (w/v) sodium chloride.

“N/A” indicates that no detectable results were obtained for this condition.

The adaptation of *A. borkumensis SK2* to low temperatures is governed by diverse mechanisms, such as the protein‐pII uridylyltransferase GlnD, which is involved in controlling the intracellular concentration of glutamine and glutamate, acting as both cryoprotectants and osmoprotectors [14, 47]. The bacterium has even systems for the synthesis and absorption of glutamate [14]. All of this information explains why the addition of glutamate to the 8× concentrates resulted in a reduction of the glass transition of 8× AB (5.9°C at 15°C/min, 7.8°C at 10°C/min, and 4.3°C at 5°C/min), while Proventus’ blend only led to a slight reduction (−2°C at 5°C/min) or even to a slight increase at 10°C/min (Table 2). Furthermore, the observed fluidizing effect is characteristic of intracellular cryoprotectants, further supporting the argument that glutamate primarily acts inside the cells [38]. Intracellular cryoprotectants, such as glutamate, act in various ways. They make the cytoplasmic membrane more plastic, bind intracellular water to prevent excessive dehydration, mitigate osmotic shocks by increasing solute concentration inside the cells, among other functions [48].

Based on two studies, the addition of bacteria to the preservation medium had no effect on the glass transition temperature, contrary to our results [37, 49]. Indeed, adding bacteria to Proventus’ blend resulted in a decrease in glass transition temperature for both amorphous phases of the solution, namely the two FCS (Table 2). However, adding bacteria to glutamate caused minimal changes in the second amorphous phase and a decrease in the glass transition temperature ranging from 0.8°C to 2.5°C for the first amorphous phase. These results suggest that the bacteria may exist in both the FCS of the Proventus’ blend and solely in the first amorphous phase with glutamate.

Therefore, to ensure a glassy state for all bacterial cells in the presence of Proventus’ blend, it is recommended that the product temperature be maintained at −59°C or below. This temperature also represents the bacterial glass transition temperature (*Tg'*) of *A. borkumensis* SK2 in the presence of Proventus' blend. For bacterial cells in the presence of glutamate 0.5 M, a temperature of −65°C or lower is recommended, as *A. borkumensis SK2* bacteria appear to be in the first amorphous phase of this solution. This temperature also represents its glass transition temperature (*Tg'*) in the presence of glutamate.

This interpretation of prokaryote vitrification integrates various theories and explanations from the literature, based on results obtained from both bacterial and mammalian cells [38, 39, 40, 45, 50, 51].

Furthermore, as previously described, it is advisable to conduct primary drying to maintain a product temperature below the cells *Tg'* with careful adjustment of the corresponding vacuum temperature and shelf temperature to ensure maintenance of a solid state. For instance, if the product in question is *A. borkumensis SK2* with 0.5 M glutamate solution, the desired product temperature would be −65°C or lower. In the presence of Proventus’ blend, it should be −59°C or lower.

These recommendations for freeze‐drying also apply to the storage of frozen formulations of *A. borkumensis SK2*. Indeed, storing formulations above *Tg'* slows bacterial metabolism without fully stopping it, allowing chemical reactions between the cells and their freeze‐concentrated environment, which increases avoidable mortality during storage [37, 38].

In this study, the freezing temperature and the primary drying parameters were set way higher than the optimal temperatures of −59°C or −65°C, depending on the cryoprotectant used. This could explain the drop in viability. As the equipment used did not allow for the measurement of product temperature, it is difficult to assess the exact state of the samples during the two freeze‐drying operations.

### 3.5 Spray‐Drying

#### 3.5.1 Effects of Spray‐Drying Parameters on Viability

**FIGURE 8:**
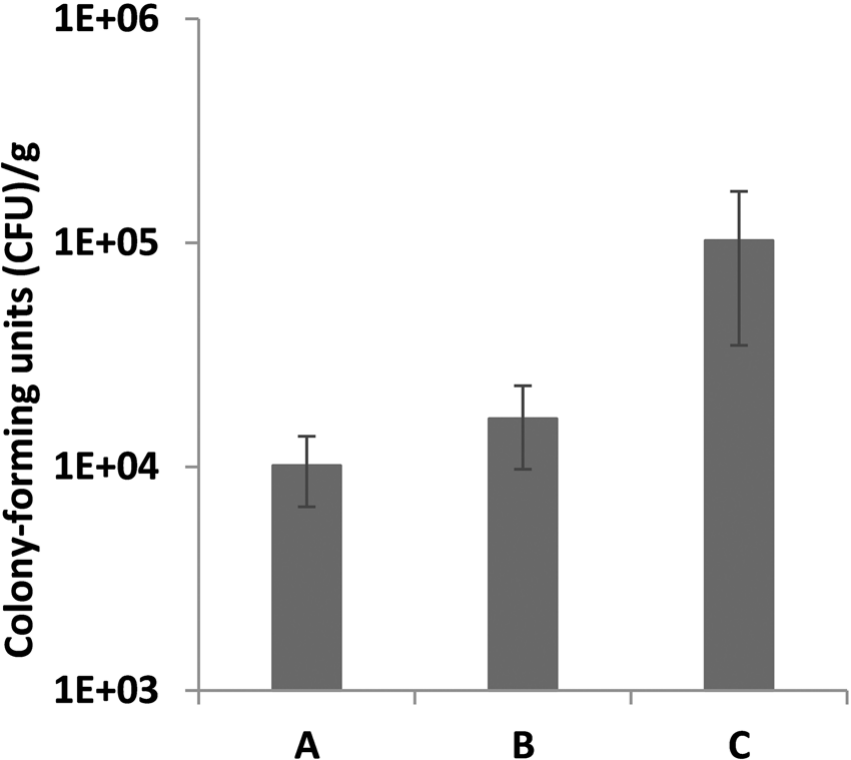
Viability of *Alcanivorax borkumensis SK2* after spray‐drying at different parameter setpoints. Parameter Set A: Inlet temperature at 150°C, pump speed at 30%, and airflow at 1758 L/h. Parameter Set B: Inlet temperature at 130°C, pump speed at 20%, and airflow at 666 L/h. Parameter Set C: Inlet temperature at 110°C, pump speed at 20%, and airflow at 1758 L/h. Common to all parameter sets: aspiration at 100%, nozzle cleaner at Level 9, final maltodextrin concentration at 15% m/v, and *A. borkumensis SK2* culture diluted 1/10. The dilution was necessary due to the type of nozzle used and the rheology of the culture. All mixtures contain the same concentration of NaCl to prevent osmotic disturbances. The results are means based on data from duplicate spray‐drying trials, and standard deviations are indicated by vertical bars.

The impact of spray‐drying on the viability of *A. borkumensis SK2* was examined. Post spray‐drying, the viability was 1.0 ± 0.4 × 10^4^, 1.6 ± 0.7 × 10^4^, 1.0 ± 0.7 × 10^5^ CFU/g for parameter sets A, B, and C, respectively (Figure 8). Parameter set C yielded the highest viability post‐spray‐drying. This observation can be attributed to the lowest inlet temperature associated with parameter set C, resulting in a lower outlet temperature and higher residual humidity, as illustrated in Figure S7. This correlation underscores the bacterium heat sensitivity, which significantly impacts cell survival during spray‐drying [52]. This sensitivity likely explains the substantial disparity in viability between spray‐drying and freeze‐drying. Freeze‐drying achieved significantly higher viability compared to spray‐drying (Figures 4, 7, and 8). The difference can be attributed not only to thermal stress but also to factors such as oxidative stress, dehydration, osmotic stress, and shear stress experienced by the bacteria during spray‐drying [53, 54].

To enhance cell survival during spray‐drying operations, exploring alternative protective agents and adjusting operational parameters, particularly outlet air temperature and feed rate, could be beneficial. The outlet air temperature is one of the key parameters influencing the viability of cultures during spray‐drying [55]. Additionally, harvesting bacteria in the stationary phase rather than during the exponential phase might also be beneficial, as demonstrated by previous studies with other bacterial strains. It has been observed, with *Lactobacillus paracasei* NFBC 338, *Lactobacillus salivarius* UCC 118, and *Lactobacillus bulgaricus*, that these bacteria exhibited less sensitivity to spray‐drying when harvested in the stationary phase [52, 56].

#### 3.5.2 Effect of Spray‐Drying Parameters on Outlet Air Temperature and Residual Humidity

The residual moisture content following spray‐drying under three different parameter sets (A, B, and C) was analyzed to evaluate the efficacy of the drying process. Samples dried under parameter sets A and B exhibited an average moisture content of 3.5% ± 0.2% and 3.5% ± 0.6%, respectively (Figure S7). Conversely, samples dried under parameter set C showed a slightly higher moisture content of 4.4% ± 0.3%. This trend is mirrored in the outlet temperatures. Samples dried under parameter sets A and B had similar outlet temperatures, being 71.5°C ± 0.7°C and 74°C ± 1°C, respectively. In contrast, samples dried under parameter Set C had a lower outlet temperature of 65°C ± 4°C, attributable to the lower inlet temperature used compared to others.

The observed moisture contents were below 5%, which aligns with the recommended residual moisture content for subsequent storage. Indeed, a residual moisture content between 3% and 5% (w/w) is recommended for the production of spray‐dried bacteria, as within this range, the resulting powders exhibit favorable characteristics, such as high flow ability, low stickiness, and maximal viability [33, 52]. Moreover, residual moisture content also impacts the rate of viability loss during storage [33].

Thus, while the current drying conditions permitted effective drying, additional adjustments to parameters or pre‐treatments appear necessary to enhance viability for spray‐drying to become an economically viable option to produce a highly viable *A. borkumensis SK2* powder. Based on our results, freeze‐drying is strongly recommended to achieve high viability of *A. borkumensis SK2* powders, enabling its commercialization and application in hydrocarbon‐contaminated marine environments.

## 4 Conclusions

*A. borkumensis SK2* has emerged as a promising tool for bioremediation due to its ability to degrade hydrocarbons effectively. The goals of this study were to identify an economically viable growth substrate for this bacterium and to develop a highly viable cell powder for its effective application in hydrocarbon‐contaminated marine environments.

The main results of this study are:

‐ Canola oil and sunflower oil are superior to the costly sodium pyruvate, yielding biomass levels (optical density) four times higher (20 ± 2 and 20 ± 1 compared to 4.6 ± 0.4, respectively).
‐ Freeze‐drying with Proventus’ blend or 0.5 M glutamate resulted in the highest cell powder viability, reaching up to 2 ± 1 × 10¹⁰ and 1.1 ± 0.3 × 10¹⁰ CFU/g after the first screening, and 1.0 ± 0.5 × 10¹⁰ and 6 ± 2 × 10⁹ CFU/g after the second, respectively.
‐ DSC analysis reveals up to 15% reduction in ice formation when increasing the cooling rate from 5°C/min to 10°C/min. In an *A. borkumensis SK2* concentrate, the 0.5 M glutamate solution reduces ice formation by up to 9% compared to Proventus’ cryoprotective blend.
‐ To promote *A. borkumensis SK2* viability during freeze‐drying, the optimal product temperatures are −65°C with 0.5 M glutamate and −59°C with Proventus’ blend.
‐ Spray‐drying produced cell powders with a viability of up to 1.0 ± 0.7 × 10⁵ CFU/g.

The novelty of this study lies in the development of a practical fermentation process to produce a highly viable bacterial powder, with the goal of achieving commercial success. More specifically, this work is the first to identify two low‐cost plant oils as sole carbon sources for *A. borkumensis SK2*, to compare spray‐drying and freeze‐drying to achieve highly viable cell powders of this bacterium, and to perform DSC analysis to define more precise freeze‐drying parameters for this strain. To our knowledge, such detailed process information is not available in public literature.

Future research should focus on further optimizing biomass production and lyophilization protocols to enhance yield and cell viability. Exploring growth temperature, trying to use a more easily assimilable nitrogen source like NH_4_^+^, and modifying the C/N ratio might promote bacterial production [24, 57].

## Author Contributions

All authors contributed to the conception and design of this study. Material preparation, data collection, and analysis were performed by É.P. The study was supervised by D.G. and P.V. The first draft of the manuscript was written by É.P. and D.G., and P.V. reviewed and edited it. All authors read and approved the final manuscript.

## Conflicts of Interest

The authors declare the following financial interests/personal relationships, which may be considered as potential competing interests: Denis Groleau reports a relationship with Proventus Bioscience Inc. that includes receiving financial compensation as a consultant.

## ChatGPT Declarations

In this article, ChatGPT 3.5 was used for the purpose of editing to improve the clarity and accuracy of the content. Specifically, ChatGPT was employed to perform reformulations to enhance the readability and comprehension of the article, while preserving the scientific integrity of the information. It is important to note that the authors supervised and carefully reviewed the modifications made by ChatGPT to ensure the consistency and scientific accuracy of the content. The use of ChatGPT was considered as an editing assistance tool, complementing the authors' work, and not as a replacement for their expertise and intellectual contribution. The authors remain fully responsible for verifying and interpreting the scientific information presented in the article.

## Supporting information




**Supporting Materials**: bab70009‐sup‐0001‐SuppMat.docx

## References

[1] “Oil Tanker Spill Statistics 2022 ,” ITOPF Ltd, 2023.

[2] R. de la Huz , M. Lastra , J. Junoy , and C. Castellanos , “Biological Impacts of Oil Pollution and Cleaning in the Intertidal Zone of Exposed Sandy Beaches: Preliminary Study of the “Prestige” Oil Spill,” Estuarine, Coastal and Shelf Science 65 (2005): 19–29, 10.1016/j.ecss.2005.03.024.

[3] M. McNutt , R. Camilli , G. Guntrie , et al., Assessment of Flow Rate Estimates for the Deepwater Horizon /Macondo Well Oil Spill (National Incident Command, Interagency Solutions Group, 2011).

[4] H. K. White , P.‐Y. Hsing , W. Cho , et al., “Impact of the Deepwater Horizon Oil Spill on a Deep‐Water Coral Community in the Gulf of Mexico,” Proceedings of the National Academy of Sciences 109 (2012): 20303–20308, 10.1073/pnas.1118029109. PMC3528508 22454495

[5] X. Yang , “Numerical Modeling of Oil Spill Containment by Boom Using SPH,” Science China Physics, Mechanics and Astronomy 56 (2013): 315–321, 10.1007/s11433-012-4980-6.

[6] V. Broje , “Improved Mechanical Oil Spill Recovery Using an Optimized Geometry for the Skimmer Surface,” Environmental Science & Technology 40 (2006): 7914–7918, 10.1021/es061842m. 17256548

[7] R. C. Prince , “A Protocol for Assessing the Effectiveness of Oil Spill Dispersants in Stimulating the Biodegradation of Oil,” Environmental Science and Pollution Research 21 (2014): 9506–9510, 10.1007/s11356-013-2053-7. 23943003 PMC4133038

[8] J. R. Bragg , R. C. Prince , and E. J. Harner , “Effectiveness of Bioremediation for the Exxon Valdez Oil Spill,” Nature 368 (1994): 413–418, 10.1038/368413a0.

[9] D. J. Naether , S. Slawtschew , S. Stasik , et al., “Adaptation of the Hydrocarbonoclastic Bacterium *Alcanivorax borkumensis SK2* to Alkanes and Toxic Organic Compounds: A Physiological and Transcriptomic Approach,” Applied and Environmental Microbiology 79 (2013): 4282–4293, 10.1128/AEM.00694-13. 23645199 PMC3697512

[10] S. Cappello , R. Denaro , M. Genovese , and L. Giuliano , “Predominant Growth of *Alcanivorax* During Experiments on “Oil Spill Bioremediation” in Mesocosms,” Microbiological Research 162 (2007): 185–190, 10.1016/j.micres.2006.05.010. 16831537

[11] A. Hara and K. Syutsubo , “ *Alcanivorax* Which Prevails in Oil‐Contaminated Seawater Exhibits Broad Substrate Specificity for Alkane Degradation,” Environmental Microbiology 5 (2003): 746–753, 10.1046/j.1468-2920.2003.00468.x. 12919410

[12] Y. Kasai , H. Kishira , T. Sasaki , K. Syutsubo , and K. Watanabe , “Predominant Growth of *Alcanivorax* Strains in Oil‐Contaminated and Nutrient‐Supplemented Sea Water,” Environmental Microbiology 4 (2002): 141–147, 10.1046/j.1462-2920.2002.00275.x. 12000314

[13] J. S. Sabirova , M. Ferrer , D. Regenhardt , and K. N. Timmis , “Proteomic Insights into Metabolic Adaptations in *Alcanivorax borkumensis* Induced by Alkane Utilization,” Journal of Bacteriology 188 (2006): 3763–3773, 10.1128/JB.00072-06. 16707669 PMC1482905

[14] S. Schneiker , V. A. P. Martins dos Santos , D. Bartels , et al., “Genome Sequence of the Ubiquitous Hydrocarbon‐Degrading Marine Bacterium *Alcanivorax borkumensis* ,” Nature Biotechnology 24 (2006): 997–1004, 10.1038/nbt1232. PMC7416663 16878126

[15] D. Sudmalis , P. Da Silva , H. Temmink , and M. M. Bijmans , “Biological Treatment of Produced Water Coupled with Recovery of Neutral Lipids,” Water Research 147 (2018): 33–42, 10.1016/j.watres.2018.09.050. 30296607

[16] B. H. Gregson , G. Metodieva , and M. V. Metodiev , “Differential Protein Expression During Growth on Linear Versus Branched Alkanes in the Obligate Marine Hydrocarbon‐Degrading Bacterium *Alcanivorax borkumensis SK2^T^ * ,” Environmental Microbiology 21 (2019): 2347–2359, 10.1111/1462-2920.14620. 30951249 PMC6850023

[17] T. Kadri , S. Magdouli , and T. Rouissi , “Ex‐situ Biodegradation of Petroleum Hydrocarbons Using *Alcanivorax borkumensis* Enzymes,” Biochemical Engineering Journal 132 (2018b): 279–287, 10.1016/j.bej.2018.01.014.

[18] M. Konieczna , M. Olzog , D. J. Naether , and Ł. Chrzanowski , “Membrane Fatty Acid Composition and Cell Surface Hydrophobicity of Marine Hydrocarbonoclastic *Alcanivorax borkumensis SK2* Grown on Diesel, Biodiesel and Rapeseed Oil as Carbon Sources,” Molecules (Basel, Switzerland) 23 (2018): 1432, 10.3390/molecules23061432. 29899233 PMC6100348

[19] M. M. Yakimov , P. N. Golyshin , S. Lang , et al., “ *Alcanivorax Borkumensis* Gen. nov., Sp. nov., a New, Hydrocarbon‐Degrading and Surfactant‐Producing Marine Bacterium,” International Journal of Systematic and Evolutionary Microbiology 48 (1998): 339–348, 10.1099/00207713-48-2-339. 9731272

[20] L. N. Warr , M. Schlüter , F. Schauer , G. M. Olson , and L. M. Basirico , “Nontronite‐Enhanced Biodegradation of Deepwater Horizon Crude Oil by *Alcanivorax borkumensis* ,” Applied Clay Science 158 (2018): 11–20, 10.1016/j.clay.2018.03.011.

[21] T. Kadri , A. Cuprys , T. Rouissi , S. K. Brar , and R. Daghrir , “Nanoencapsulation and Release Study of Enzymes from *Alkanivorax borkumensis* in Chitosan‐tripolyphosphate Formulation,” Biochemical Engineering Journal 137 (2018a): 1–10, 10.1016/j.bej.2018.05.013.

[22] T. Kadri , S. Miri , T. Robert , et al., “Pilot‐Scale Production and In‐Situ Application of Petroleum‐Degrading Enzyme Cocktail From *Alcanivorax borkumensis* ,” Chemosphere 295 (2022): 133840, 10.1016/j.chemosphere.2022.133840. 35124086

[23] L. Lonappan , T. Guedri , T. Rouissi , and S. K. Brar , “Chlorpyrifos Degradation by Crude Enzyme Extracts Obtained from *Alcanivorax borkumensis* ,” Integrated and Sustainable Environmental Remediation (American Chemical Society, 2018), 81–95.

[24] T. Karmainski , M. R. E. Dielentheis‐Frenken , M. K. Lipa , A. N. T. Phan , and L. M. Blank , “High‐quality Physiology of *Alcanivorax borkumensis SK2* Producing Glycolipids Enables Efficient Stirred‐Tank Bioreactor Cultivation,” Frontiers in Bioengineering and Biotechnology 11 (2023), 10.3389/fbioe.2023.1325019. PMC10710537 38084272

[25] S. S. Radwan , M. M. Khanafer , and A.l‐A. HA , “Ability of the So‐Called Obligate Hydrocarbonoclastic Bacteria to Utilize Nonhydrocarbon Substrates Thus Enhancing Their Activities Despite Their Misleading Name,” BMC Microbiology 19 (2019): 41, 10.1186/s12866-019-1406-x. 30777002 PMC6379940

[26] M. Omarova , L. T. Swientoniewski , I. K. Mkam Tsengam , et al., “Biofilm Formation by Hydrocarbon‐Degrading Marine Bacteria and Its Effects on Oil Dispersion,” ACS Sustainable Chemistry and Engineering 7 (2019): 14490–14499, 10.1021/acssuschemeng.9b01923.

[27] A. Sánchez , R. Maceiras , and A. Cancela , “Influence of *n*‐Hexane on *in Situ* Transesterification of Marine Macroalgae,” Energies 5 (2012): 243–257, 10.3390/en5020243.

[28] N. Boz , “Solid Base Catalyzed Transesterification of Canola Oil,” Chemical Engineering Communications 196 (2008): 80–92, 10.1080/00986440802301438.

[29] Selina Wamucii , “Canada Canola Oil Prices,” Selina Wamucii, 2024, https://www.selinawamucii.com/insights/prices/canada/canola‐oil/.

[30] Selina Wamucii , “Canada Sunflower Oil Prices,” Selina Wamucii, 2024, https://www.selinawamucii.com/insights/prices/canada/sunflower‐oil/.

[31] A2B Chem LLC , “Sodium Pyruvate (Cas: 113‐24‐6) 99% Quote, 1 Ton,” A2B Chem LLC, 2024.

[32] Z. Zhang , Y. Yu , Y. Wang , et al., “Development of a New Protocol for Freeze‐Drying Preservation of *Pseudoalteromonas nigrifaciens* and Its Protective Effect on Other Marine Bacteria,” Electronic Journal of Biotechnology 44 (2020): 1–5, 10.1016/j.ejbt.2019.12.006.

[33] G. Zayed , “Influence of Trehalose and Moisture Content on Survival of *Lactobacillus salivarius* Subjected to Freeze‐Drying and Storage,” Process Biochemistry 39 (2004): 1081–1086, 10.1016/S0032-9592(03)00222-X.

[34] N. Ekdawi‐Sever and L. a. Goentoro , “Effects of Annealing on Freeze‐Dried *Lactobacillus acidophilus* ,” Journal of Food Science 68 (2003): 2504–2511, 10.1111/j.1365-2621.2003.tb07052.x.

[35] H. J. Jeon , J. Kim , W. Y. Seok , et al., “Changes in the Metabolome of Probiotics During the Stationary Phase Increase Resistance to Lyophilization,” Food Bioscience 53 (2023): 102499, 10.1016/j.fbio.2023.102499.

[36] J. S. Sabirova , A. Becker , H. Lünsdorf , J.‐M. Nicaud , and K. N. Timmis , “Transcriptional Profiling of the Marine Oil‐degrading Bacterium *Alcanivorax borkumensis* During Growth on *n*‐Alkanes,” FEMS Microbiology Letters 319 (2011): 160–168, 10.1111/j.1574-6968.2011.02279.x. 21470299

[37] K. S. Pehkonen , Y. H. Roos , S. Miao , and R. P. Ross , “State Transitions and Physicochemical Aspects of Cryoprotection and Stabilization in Freeze‐drying of *Lactobacillus rhamnosus GG* (LGG),” Journal of Applied Microbiology 104 (2008): 1732–1743, 10.1111/j.1365-2672.2007.03719.x. 18248378

[38] F. Fonseca , J. Meneghel , S. Cenard , and S. Passot , “Determination of Intracellular Vitrification Temperatures for Unicellular Micro Organisms under Conditions Relevant for Cryopreservation,” PLOS One 11 (2016): e0152939, 10.1371/journal.pone.0152939. 27055246 PMC4824440

[39] A. Hauptmann and G. Hoelzl , “Optical Cryomicroscopy and Differential Scanning Calorimetry of Buffer Solutions Containing Cryoprotectants,” European Journal of Pharmaceutics and Biopharmaceutics 163 (2021): 127–140, 10.1016/j.ejpb.2021.03.015. 33813056

[40] C. Santivarangkna , M. Aschenbrenner , and U. Kulozik , “Role of Glassy State on Stabilities of Freeze‐Dried Probiotics,” Journal of Food Science 76 (2011): R152–R156, 10.1111/j.1750-3841.2011.02347.x. 22417602

[41] H. Kiani , “Water Crystallization and Its Importance to Freezing of Foods: A Review,” Trends in Food Science & Technology 22 (2011): 407–426, 10.1016/j.tifs.2011.04.011.

[42] W. Q. Sun , “Calorimetric Analysis of Cryopreservation and Freeze‐Drying Formulations,” in Cryopreservation and Freeze‐Drying Protocols, ed. W. F. Wolkers , H. Oldenhof (Springer, 2015), 163–179. 10.1007/978-1-4939-2193-5_6 25428006

[43] S. Yakovlev , “Crystalline Ice as a Cryoprotectant: Theoretical Calculation of Cooling Speed in Capillary Tubes,” Journal of Microscopy 243 (2011): 8–14, 10.1111/j.1365-2818.2011.03498.x. 21534954 PMC4189803

[44] A. Bogdan , M. J. Molina , H. Tenhu , E. Bertel , and N. Bogdan , “Visualization of Freezing Process *in Situ* Upon Cooling and Warming of Aqueous Solutions,” Scientific Reports 4 (2014): 7414, 10.1038/srep07414. 25491562 PMC4261172

[45] F. Fonseca and M. Marin , “Stabilization of Frozen *Lactobacillus delbrueckii* Subsp. *bulgaricus* in Glycerol Suspensions: Freezing Kinetics and Storage Temperature Effects,” Applied and Environmental Microbiology 72 (2006): 6474–6482, 10.1128/AEM.00998-06. 17021195 PMC1610330

[46] V. M. Odagescu , “ Études liées à la vitrification sans fracture de solutions cryoprotectrices,” (thesis, Université Joseph‐Fourier—Grenoble I, 2005).

[47] J. S. Sabirova , T. N. Chernikova , and K. N. Timmis , “Niche‐Specificity Factors of a Marine Oil‐Degrading Bacterium *Alcanivorax borkumensis SK2* ,” FEMS Microbiology Letters 285 (2008): 89–96, 10.1111/j.1574-6968.2008.01222.x. 18557784

[48] Z. Hubálek , “Protectants Used in the Cryopreservation of Microorganisms,” Cryobiology 46 (2003): 205–229, 10.1016/S0011-2240(03)00046-4. 12818211

[49] Å. Schoug , J. Olsson , J. Carlfors , and J. Schnürer , “Freeze‐Drying of *Lactobacillus coryniformis* Si3—Effects of Sucrose Concentration, Cell Density, and Freezing Rate on Cell Survival and Thermophysical Properties,” Cryobiology 53 (2006): 119–127, 10.1016/j.cryobiol.2006.04.003. 16756971

[50] A. Clarke , G. J. Morris , F. Fonseca , B. J. Murray , and E. Acton , “A Low Temperature Limit for Life on Earth,” PLOS One 8 (2013): e66207, 10.1371/journal.pone.0066207. 23840425 PMC3686811

[51] G. John Morris , E. Acton , and B. J. Murray , “Freezing Injury: The Special Case of the Sperm Cell,” Cryobiology 64 (2012): 71–80, 10.1016/j.cryobiol.2011.12.002. 22197768

[52] G. E. Gardiner , E. O'Sullivan , J. Kelly , et al., “Comparative Survival Rates of Human‐Derived Probiotic *Lactobacillus paracasei* and *L. salivarius* Strains during Heat Treatment and Spray Drying,” Applied and Environmental Microbiology 66 (2000): 2605–2612, 10.1128/AEM.66.6.2605-2612.2000. 10831444 PMC110587

[53] G. Broeckx , D. Vandenheuvel , I. J. J. Claes , and S. Lebeer , “Drying Techniques of Probiotic Bacteria as an Important Step Towards the Development of Novel Pharmabiotics,” International Journal of Pharmaceutics 505 (2016): 303–318, 10.1016/j.ijpharm.2016.04.002. 27050865

[54] G. Broeckx , D. Vandenheuvel , T. Henkens , et al., “Enhancing the Viability of *Lactobacillus Rhamnosus* GG After Spray Drying and during Storage,” International Journal of Pharmaceutics 534 (2017): 35–41, 10.1016/j.ijpharm.2017.09.075. 28986319

[55] E. Ananta and M. Volkert , “Cellular Injuries and Storage Stability of Spray‐Dried *Lactobacillus Rhamnosus* GG,” International Dairy Journal 15 (2005): 399–409, 10.1016/j.idairyj.2004.08.004.

[56] P. Teixeira and H. Castro , “Spray Drying as a Method for Preparing Concentrated Cultures of *Lactobacillus bulgaricus* ,” Journal of Applied Bacteriology 78 (1995): 456–462, 10.1111/j.1365-2672.1995.tb03433.x.

[57] T. Karmainski , M. K. Lipa , S. Kubicki , et al., “Optimized Feeding Strategies for Biosurfactant Production from Acetate by *Alcanivorax Borkumensis SK2* ,” Fermentation 10 (2024): 257, 10.3390/fermentation10050257.

