# Supplementary material for: Production and Formulation of Alcanivorax borkumensis SK2 Cell Powders for Marine Oil Spill Bioremediation
Source: Biotechnol Appl Biochem. 2025 Jul 7;73(1):123–37. doi: 10.1002/bab.70009 (PMC12902735; doi:10.1002/bab.70009)
Supplement: Supplementary file 1 — Supporting Materials: bab70009‐sup‐0001‐SuppMat.docx [file BAB-73-123-s001.docx]

**Supporting Information**

**Production and Formulation of *Alcanivorax borkumensis SK2* Cell Powders**

**for Marine Oil Spill Bioremediation**

*Biotechnology and Applied Biochemistry*

Élisabeth Perreault^1^, Denis Groleau^2^, Patrick Vermette^1*^

1. Laboratoire de bio-ingénierie et de biophysique de l’Université de Sherbrooke, Department of Chemical and Biotechnological Engineering, Université de Sherbrooke, 2500 boul. de l’Université, Sherbrooke, QC, Canada, J1K 2R1.
2. Department of Chemical and Biotechnological Engineering, Université de Sherbrooke, 2500 boul. de l’Université, Sherbrooke, QC, Canada, J1K 2R1.

*Corresponding author: Department of Chemical and Biotechnological Engineering,

Université de Sherbrooke, 2500 boul. de l’Université, Sherbrooke, Québec, Canada, J1K 2R1.

Phone: 1-819-821-8000 ext. 62826; E-mail: Patrick.Vermette@USherbrooke.ca

**Freezingx**

Fig. S1 Freeze-drying program used for *Alcanivorax borkumensis SK2* cell powder production. Freezing phase: 25 °C to -30 °C at 1013.25 mbar, held for 16 h. Primary drying phase: stage 1 at -35 °C, 0.127 mbar, 0.5 h; stage 2 at -35 °C, 0.127 mbar, 0.67 h; stage 3 at 10 °C, 0.146 mbar, 15 h; stage 4 at 15 °C, 0.146 mbar, 1.67 h; stage 5 at 25 °C, 0.146 mbar, 4 h; stage 6 at 30 °C, 0.133 mbar, 20.83 h; stage 7 at 30 °C, 0.133 mbar, 6 h. Secondary drying phase: stage 1 at 30 °C, 0.05 mbar, 0.17 h; stage 2 at 30 °C, 0.05 mbar, 5 h. All data correspond to a single freeze-drying program.

Fig. S2 Residual moisture of *A. borkumensis SK2* formulations after freeze-drying with different cryoprotectants at concentrations of 0.05 M (C1) and 0.5 M (C2) compared to Proventus’s cryoprotectant blend. The results represent the means obtained from duplicates with standard deviations indicated by vertical bars.


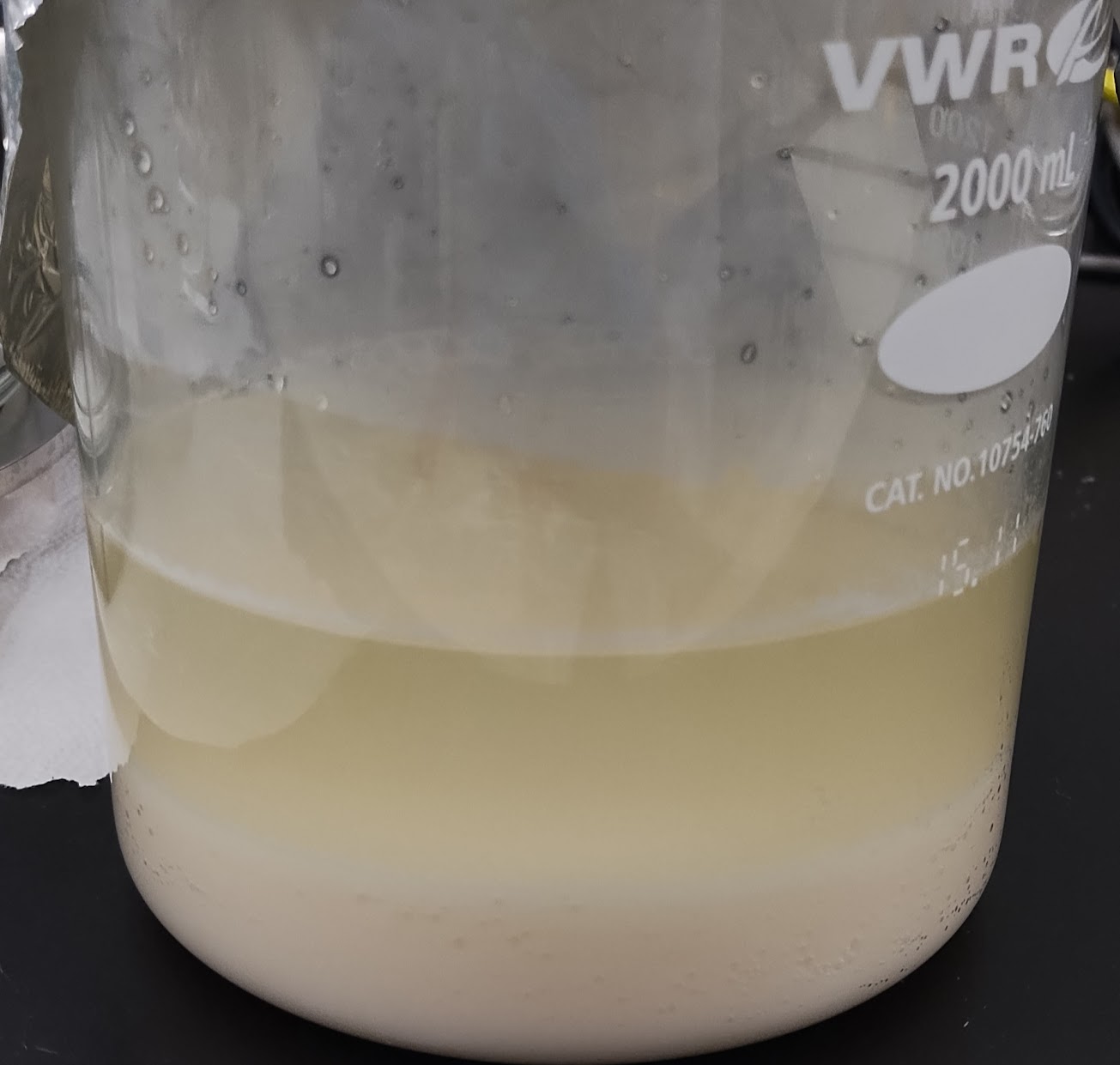


Fig. S3 Highly hydrophobic sedimenting concentrate from Fermentation No.2.

Fig. S4 Residual moisture of *A. borkumensis SK2* formulations after freeze-drying with glutamate at concentrations of 0.05 M (C1), 0.275 M (C2), and 0.5 M (C3) compared to Proventus’s cryoprotectant blend. The results are obtained from only one of the duplicates to preserve the sterility of the other duplicate by minimizing its handling, due to the contamination risk observed in recent lab activities.

Fig. S5 Analytical DSC profiles during cooling (upper solid line curve) from 20 to -80 °C and subsequent heating (lower solid line) from -80 to 20 °C of an 8X AB sample. First derivative of the heating curve showed as the dotted line. Scanning rate: 10 °C/min.

Table S1 Melting temperature shift and crystallinity loss of a *A. borkumensis SK2* 8X concentrate in the presence and absence of cryoprotectants, as well as cryoprotectants alone, under various freezing rates.

| Formulation | Freezing rate | Melting temperature reduction (Onset) (℃) | Crystallinity loss** (%) | Delta*** Crystallinity loss  (%) |
| --- | --- | --- | --- | --- |
| Distilled water  VS  Prov* | 15 ℃/min | 7.1 | 15 |  |
|  | 10 ℃/min | 4.7 | 15 | 1 |
|  | 5 ℃/min | 5.6 | 1 | 14 |
| Distilled water  VS  Prov + 8X AB* | 15 ℃/min | 7.4 | 27 |  |
|  | 10 ℃/min | 5.0 | 27 | 0 |
|  | 5 ℃/min | 5.7 | 14 | 13 |
| Distilled water  VS  Glu* | 15 ℃/min | 7.4 | 23 |  |
|  | 10 ℃/min | 5.0 | 22 | 1 |
|  | 5 ℃/min | 5.7 | 10 | 12 |
| Distilled water  VS  Glu + 8X AB* | 15 ℃/min | 10.8 | 32 |  |
|  | 10 ℃/min | 8.7 | 32 | 1 |
|  | 5 ℃/min | 9.1 | 23 | 9 |
| Distilled water  VS  8X AB* | 15 ℃/min | 6.5 | 27 |  |
|  | 10 ℃/min | 4.4 | 28 | -1 |
|  | 5 ℃/min | 5.2 | 14 | 14,5 |

****:** The percentage of crystallinity was calculated by the ratio of the specific corrected melting enthalpy to the specific melting enthalpy of distilled water. The specific corrected melting enthalpy is the specific melting enthalpy adjusted to account for the exact weight of water in each sample, rather than the weight of the entire sample, as only water undergoes crystallization. It was calculated assuming that the 8X concentrate of *Alcanivorax borkumensis SK2* was entirely composed of water, as the exact composition is unknown.

**Example of calculation for** ${\boldsymbol{Crystallinity loss}_{\boldsymbol{Distilled water VS Glu}}}_{\boldsymbol{15℃/min}}$**:**

$Crystallinity loss=distilled water crystallinity-Glu crystallinity$

$$Crystallinity loss=100 \%-{crystallinity}_{15℃/min}$$

$Crystallinity loss=100 \%-\frac{specific corrected melting enthalpy}{specific melting enthalpy of distilled water}$

$Crystallinity loss=100 \% \left( \frac{\frac{specific melting enthalpy}{\left( mass fraction of water +mass fraction of AB concentrate \right)}}{336 J/g} \right)*100\%$

$Crystallinity loss=100 \%-\frac{\frac{234 J/g}{\left( 0.90+0 \right)}}{336J/g}*100\%$

$Crystallinity loss=23.619\%$

$Crystallinity loss=23\%$

*****:** For each comparison of crystallinity loss under different freezing rates, the calculation involves: Subtracting the value at 10 °C/min from the value at 15 °C/min, or subtracting the value at 5 °C/min from the value at 10 °C/min.

**Example:**

${Delta Crystallinity loss}^{***}{{=\% Crystallinity loss}_{Distalled water VS Prov+8X AB}}_{10℃/min-}$

${{\% Crystallinity loss}_{Distalled water VS Prov+8X AB}}_{5℃/min}$

${Delta Crystallinity loss}^{***}= 15\%-14\%$

${Delta Crystallinity loss}^{***}= 1\%$

Table S2 Comparison of thermal properties during heating of a *A. borkumensis SK2* 8X concentrate in the presence and absence of cryoprotectants, as well as cryoprotectants alone, under various freezing rates.

| Formulation | Freezing rate | Melting temperature reduction (Onset) (℃) | Crystallinity loss** (%) | Relative variation of corrected specific enthalpy loss ****  % |
| --- | --- | --- | --- | --- |
| 8X AB*  VS  Prov+8X AB* | 15 ℃/min | 0.8 | 0% | 0% |
|  | 10 ℃/min | 0.6 | -1% | -1% |
|  | 5 ℃/min | 0.5 | 1% | 1% |
| 8X* AB  VS  Glu+8X AB* | 15 ℃/min | 4.2 | 5% | 7% |
|  | 10 ℃/min | 4.3 | 4% | 5% |
|  | 5 ℃/min | 3.9 | 9% | 11% |
| Glu+8X* AB  VS  Glu * | 15 ℃/min | -0.3 | -10% | -15% |
|  | 10 ℃/min | -0.5 | -10% | -15% |
|  | 5 ℃/min | 0.1 | -13% | -17% |
| Prov+8X* AB  VS  Prov* | 15 ℃/min | -0.3 | -12% | -17% |
|  | 10 ℃/min | -0.3 | -13% | -17% |
|  | 5 ℃/min | -0.1 | -13% | -16% |

****: Calculated assuming that the 8X concentrate of *Alcanivorax borkumensis SK2* was entirely composed of water and adjusting the specific enthalpy as if the sample was 100% water.

Fig. S6 Analytical DSC profiles during cooling (upper solid line curve) from 20 to -80 °C and subsequent heating (lower solid line) from -80 to 20 °C of an 8X AB + Glu sample. First derivative of the heating curve shown as the dotted line. Scanning rate: 10 °C/min.

Fig. S7 Residual moisture of *A. borkumensis SK2* formulations after spray-drying at different parameter sets. Parameter set A: Inlet temperature at 150 ℃, pump speed at 30%, and airflow at 1758 L/h. Parameter set B: Inlet temperature at 130 ℃, pump speed at 20%, and airflow at 666 L/h. Parameter set C: Inlet temperature at 110 ℃, pump speed at 20%, and airflow at 1758 L/h. Common to all parameter sets: aspiration at 100%, nozzle cleaner at level 9, final maltodextrin concentration at 15% m/v, and *A. borkumensis SK2* culture diluted 1/10. The dilution was necessary due to the type of nozzle used and the rheology of the culture. All mixtures contain the same concentration of NaCl to prevent osmotic disturbances. The line shows the outlet temperature of the corresponding powders. The results are means based on data from duplicate spray-drying trials, and standard deviations are indicated by vertical bars.
